# Research Progress on the Preparation Methods for and Flame Retardant Mechanism of Black Phosphorus and Black Phosphorus Nanosheets

**DOI:** 10.3390/nano14100892

**Published:** 2024-05-20

**Authors:** Wuyan Cao, Dengwang Lai, Jun Yang, Li Liu, Hao Wu, Jin Wang, Yuejun Liu

**Affiliations:** 1 Key Laboratory of Advanced Packaging Materials and Technology of Hunan Province, Hunan University of Technology, Zhuzhou 412007, China; m22080500004@stu.hut.edu.cn (W.C.); m23080500012@stu.hut.edu.cn (L.L.); m21080500014@stu.hut.edu.cn (H.W.); liuyuejun@hut.edu.cn (Y.L.); 2 Zhuzhou Times New Material Technology Co., Ltd., Zhuzhou 412007, China; wangjin@csrzic.com

**Keywords:** BP, BPNSs, flame retardant

## Abstract

Black phosphorus and black phosphorus nanosheets are widely used in the flame retardant field because of their excellent properties, but the immature preparation methods have resulted in extremely high preparation cost, which greatly limits their development and application. In this paper, various preparation methods of black phosphorus and black phosphorus nanosheets are described in detail, the advantages and disadvantages of each method are analyzed in depth, the flame-retardant mechanism and application of black phosphorus and black phosphorus nanosheets in flame retardants are discussed, and the subsequent development direction of black phosphorus and black phosphorus nanosheets is proposed.

## 1. Introduction

According to the 2022 China Phosphorus Chemical Industry Market Outlook and Investment Strategy Planning Analysis Report, China is the world’s largest producer and consumer of phosphorus chemical products. In 2021, China produced 777,500 tonnes of yellow phosphorus and consumed about 750,000 tonnes, both figures lower than those in 2016 [1]. China’s phosphorus chemical industry is currently facing a bottleneck, despite being largely self-sufficient, and diversification, personalization, and high-end products are the prevailing trends [2]. Among them, black phosphorus (BP) has garnered significant attention due to its exceptional performance.

Black phosphorus (BP) is an allotrope of phosphorus, along with red phosphorus (RP), white phosphorus (WP), and violet phosphorus. Black phosphorus nanosheets (BPNSs) have a layer-like structure very similar to graphene, with layers connected by weak van der Waals forces [3,4]. Black phosphorus nanosheets have a unique wrinkled honeycomb structure, which gives them many outstanding properties, such as excellent optoelectronic performance, high charge carrier mobility, field-effect transistor effects, and high theoretical specific capacity. This has led to wide applications of black phosphorus nanosheets in optoelectronics [5], energy storage [6,7], sensors [8,9], batteries [10,11], and other fields. In recent years, it has also been reported that black phosphorus nanosheets have tremendous potential for development in the field of flame retardancy, significantly increasing the limiting oxygen index (LOI) and reducing the peak heat release rate (PHRR) of materials at low addition levels (mass fraction ≤ 10%).

However, the promising application prospects of black phosphorus nanosheets are hindered by its demanding preparation methods. Currently, the preparation of black phosphorus nanosheets mostly relies on black phosphorus as the raw material. However, the preparation of black phosphorus itself faces issues such as high cost and difficulty in scaling up, resulting in products with more or less impurities and poor crystallinity. The price of black phosphorus on the market even exceeds that of gold [12,13,14]. Low-cost and scalable production of black phosphorus nanosheets is currently a pressing issue, which is of great importance for promoting the application of black phosphorus nanosheets. Therefore, this paper elaborates on the preparation methods of black phosphorus and black phosphorus nanosheets, summarizes the advantages and disadvantages of each method, and analyzes and prospects the application prospects of both.

## 2. Preparation of Black Phosphorous

Currently, there are many methods to prepare black phosphorus, which can be broadly classified into the pressurized method and the catalytic method according to the preparation principle. Pressurization refers to the preparation of black phosphorus by a phase change of red or white phosphorus through high pressure. Methods such as the high temperature and high pressure method and the mechanical ball milling method are categorized as high-pressure methods. The catalytic method refers to reducing the activation energy of the reaction by adding a catalyst so that black phosphorus can be prepared under lower pressure. Examples of the catalytic method include the mercury reflux method, bismuth melting method, and mineralization method.

### 2.1. Pressurization

#### 2.1.1. High Temperature and High Pressure Method

The high temperature and high pressure method is a widely used technique for preparing black phosphorus crystals under specific conditions. The initial production of black phosphorus dates back to the early 20th century, when Bridgman successfully converted white phosphorus into black phosphorus using high-temperature and high-pressure conditions (1.2–1.3 GPa hydrostatic pressure, 200 °C). Subsequently, Bridgman explored the production of bulk black phosphorus at lower temperatures [15], resulting in a method that yielded superior quality and quantity compared to the previous approach, albeit requiring higher pressure (8.0 GPa). Despite the harsh preparation conditions, this method stands out as one of the most effective for preparing black phosphorus. Moreover, scholars have continuously refined this approach over time. Jacobs [16] successfully prepared black phosphorus at hydrostatic pressures ranging from 1.1 Gpa to 1.6 Gpa. Shirotani [17] synthesized nano-sized crystals of black phosphorus at 2.3 Gpa and 500 °C. Akahama [18] produced bulk black phosphorus at 1 Gpa and 900 °C. Sun et al. [19] rapidly prepared bulk black phosphorus at temperatures between 20 °C and 800 °C under pressures of 2–5 Gpa. Zhao et al. [20] produced black phosphorus materials doped with transition metals at 1.6 Gpa and 700 °C. However, the harsh conditions required to achieve a high temperature and high pressure make this method unsuitable for large-scale production.

#### 2.1.2. Mechanical Ball Milling

**Figure 1:**
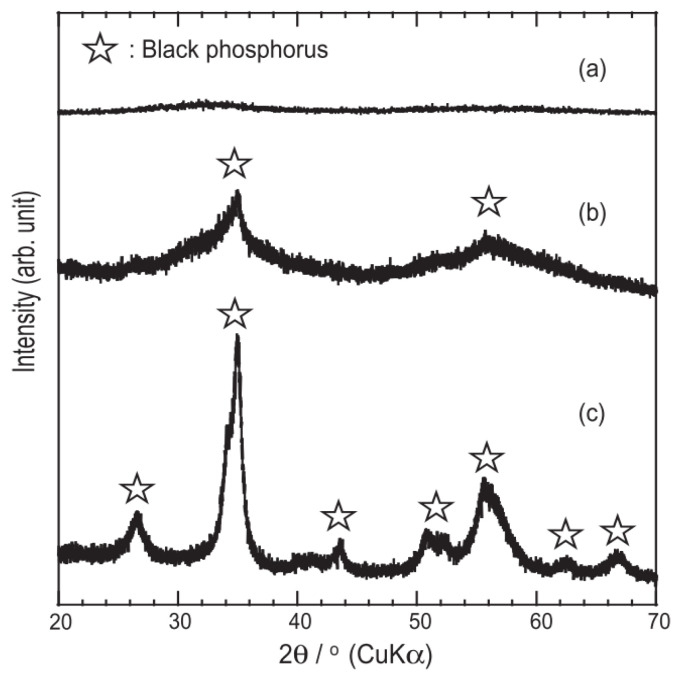
XRD plots of each material: (**a**) RP; (**b**) BP made by a planetary ball mill; (**c**) BP made by a hybrid ball mill [23]. Reprinted with permission from Ref. [23]. Copyright 2010, copyright Motohiro Nagao.

The mechanical ball milling method involves using ball milling media to impact RP at a high speed in a ball mill to produce black phosphorus. The fundamental principle of the ball milling method is akin to that of the high temperature and high pressure method. It also utilizes high-speed impact to generate high pressure, inducing a phase change in RP. As early as the last century, Günther et al. [21] employed mechanical ball milling to synthesize bulk black phosphorus. Due to black phosphorus’s high susceptibility to oxidation, Park et al. [22] used the inert gas argon to fill the environment during preparation. Nagao et al. [23] compared the X-ray diffraction patterns (XRD) of black phosphorus and found that black phosphorus prepared by a hybrid ball mill exhibits superior crystallinity compared to that prepared by a planetary ball mill (Figure 1). However, the ball milling method for obtaining the BP crystal form is less effective than the high temperature and high pressure method. Following this, some scholars utilized the ball milling method to prepare black phosphorus composite materials, thus laying the groundwork for the preparation of black phosphorene.

However, the BP crystal form produced by mechanical ball milling is of poor quality and requires an extended processing time, often spanning dozens of hours. This extended processing time significantly increases costs, rendering it unsuitable for large-scale applications.

### 2.2. Catalytic Methods

#### 2.2.1. The Mercury Reflux Method and the Bismuth Melting Method

The mercury catalytic method utilizes mercury to lower the activation energy, making it a form of catalytic method in theory. Kerbs et al. [24] used metallic mercury to reduce the pressure during the reaction and prepared millimeter-sized massive black phosphorus at 370–410 °C. Despite requiring a lower pressure, this method has a lengthy experimental cycle and poses health risks due to the use of metallic mercury. Furthermore, the process of removing residual mercury also reduces production efficiency. The bismuth fusion method bears some resemblance to the mercury reflux method. Baba et al. [25] initially synthesized white phosphorus from red phosphorus as the raw material under inert gas protection. Subsequently, they reacted white phosphorus with hot bismuth at 400 °C to obtain BP crystals. However, the white phosphorus used in the experiment, along with the nitric acid solution used in the subsequent washing process, is hazardous. Moreover, the product contains numerous sulfides and other impurities.

Based on the experimental results of numerous scholars, both the mercury reflux method and the bismuth fusion method exhibit several disadvantages. These methods employ inherently hazardous raw materials, yield a low output, and incur high experimental costs. Consequently, few researchers choose to utilize these two methods for black phosphorus preparation.

#### 2.2.2. Mineralization

**Figure 2:**
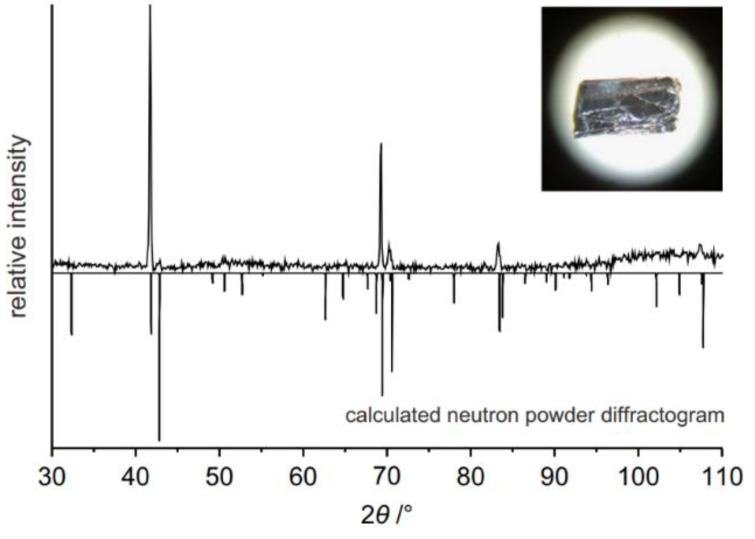
Neutron diffraction pattern of black phosphorus [28]. Reprinted with permission from Ref. [28]. Copyright 2014, copyright Marianne Köpf.

**Figure 3:**
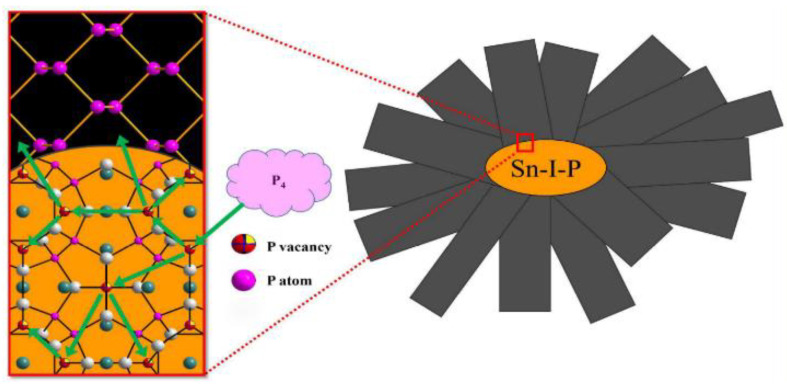
Li’s hypothesized growth machine [29]. Reprinted with permission from Ref. [29]. Copyright 2018, copyright Sheng Li.

While all the aforementioned methods can produce bulk black phosphorus, the high pressure method necessitates an exceedingly high pressure. Additionally, the experimental conditions of the ball milling method, mercury reflux method, and bismuth fusion method are relatively stringent, significantly impeding black phosphorus production. To address these challenges, scholars have discovered a new preparation method, the mineralization method, through extensive experimental studies. The mineralization method utilizes safer red phosphorus as a raw material, which is then mixed with a mineralizing agent in specific proportions. Subsequently, the mixture undergoes a series of temperature treatments, including heating and cooling, to produce well-crystallized bulk black phosphorus. Lange et al. [26] utilized this method to prepare bulk black phosphorus by mixing Au, Sn, and SnI4 as mineralizers with red phosphorus and conducting the reaction at 600 °C. Nilges et al. [27] refined their experimental approach by placing the mineralizing agent into a sealed quartz tube and subjecting it to controlled temperature changes. The process took more than 30 h to produce black phosphorus. Subsequent researchers further optimized the mineralizer to lower experimental costs. Köpf et al. [28] exclusively employed Sn and SnI4 as mineralizers, and successfully produced centimeter-scale bulk black phosphorus by adjusting the reaction temperature. The neutron powder diffraction pattern (Figure 2) reveals the presence of only one crystal form of black phosphorus throughout the entire reaction process. Li et al. [29] conducted extensive studies and experiments on the formation temperature and growth model of black phosphorus. They proposed a new “gas–solid–solid” phase growth mechanism, as illustrated in Figure 3. Through experiments, they found that Sn_24_P_22_-_X_I_8_ is relatively stable below 520 °C, when P_4_ can break down on its surface and diffuse into it. When the concentration of phosphorus is high enough, it will precipitate on the surface of Sn_24_P_22_-_X_I_8_ to form black phosphorus. The morphology of the black phosphorus bands is uniform, which satisfies the “gas–solid–solid” phase growth mechanism proposed by them.

The development of high pressure methods for preparing black phosphorus has raised prospects for large-scale production. Presently, researchers are continuing to explore methods to reduce experimental costs, focusing on raw materials, reaction mechanisms, and other aspects. Furthermore, the formation mechanism of black phosphorus has long been a topic of controversy. The current explanations encompass two mechanisms: the “gas–solid–solid” phase and phase induction. Our goal remains to achieve the high-quality, low-cost, and large-scale production of black phosphorus.

### 2.3. Comparison of Various Preparation Methods

The advantages and disadvantages of various preparation methods for black phosphorus are shown in Table 1.

## 3. Preparation of Black Phosphorus Nanosheets

At present, the methods for preparing BPNSs can be broadly classified into two categories: the “top–down method” and the “bottom–up method”. The former involves producing black phosphorus nanosheets through the exfoliation of large pieces of black phosphorus crystals using external forces such as ultrasonic waves and electric fields. This process generally includes liquid phase exfoliation and mechanical exfoliation. Various liquid phase exfoliation methods exist. These include the electrochemical exfoliation method, ultrasonic exfoliation method, shear exfoliation method, microwave exfoliation method, etc. It is noteworthy that ultrasonic exfoliation is a method widely used by industry, academia, and research institutes for preparing black phosphorus nanosheets. The latter refers to the preparation of black phosphorene through the incremental accumulation of molecules or atoms. The commonly used methods include the solvothermal method, chemical vapor deposition method, and high pressure method.

### 3.1. Top–Down Method

#### 3.1.1. Mechanical Stripping Method

**Figure 4:**
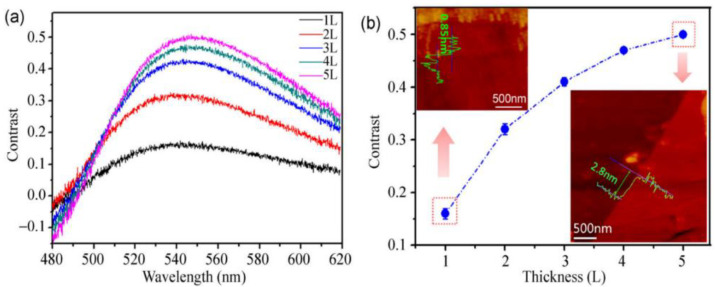
(**a**) Contrast spectra of phosphenes with different number of layers. (**b**) Contrast values of 1–5 layers of phosphene (inset: corresponding AFM images from two phosphene flakes with different optical contrasts) [34]. Reprinted with permission from Ref. [34]. Copyright 2014, copyright Wanglin Lu.

The mechanical exfoliation method is commonly employed to isolate two-dimensional layered materials into nanomaterials with a few layers or a single atomic layer thickness. In 2004, Novoselov et al. [30] demonstrated the mechanical exfoliation method by using tape to isolate a small amount of single-layer graphene from bulk graphite. The structure of black phosphorus nanosheets closely resembles that of graphite, as both materials exhibit a layered structure held together by weak van der Waals forces between the layers. Consequently, some scholars have explored the use of this method to prepare black phosphorus nanosheets. Li et al. [31] successfully employed tape to peel off black phosphorus nanosheets. Additionally, they affixed the black phosphorus flakes to silicon dioxide silicon wafers to fabricate field-effect transistors (FETs) for microprocessors and memories. Chen et al. [32] similarly employed tape to peel off black phosphorus films. However, this method results in a very low yield of black phosphorus nanosheets, and both black phosphorus and black phosphorus nanosheets are prone to oxidation, leading to low product purity. Subsequently, some scholars enhanced the tape exfoliation method. Castellanos-Gomez et al. [33] augmented this approach by incorporating a silicone resin transmission layer, thereby enhancing the yield of black phosphorus nanosheets. Lu [34] combined Ar^+^ plasma with the tape exfoliation method to produce more stable black phosphorus nanosheets. Extensive characterization revealed that the structure of the black phosphorene was intact and not oxidized, as shown in Figure 4.

Scholars have successfully demonstrated the feasibility of preparing black phosphorus nanosheets through mechanical exfoliation. While the experimental conditions are simple, the process requires a significant amount of time and intensive labor. Moreover, the yields of various improved methods are not consistent [35,36]. Furthermore, the challenge of raw material oxidation remains unsolved. The introduction of inert gas only partially mitigates the oxidation of black phosphorus and black phosphorene, resulting in a final product that is still partially oxidized.

#### 3.1.2. Liquid Phase Exfoliation

The liquid phase exfoliation method is a widely employed technique for preparing nanomaterials. Due to its high yield, good quality, and minimal structural damage to BPNSs, the liquid phase exfoliation method is one of the most commonly employed techniques for preparing BPNSs. BPNSs are prepared by dispersing bulk black phosphorus or black phosphorus powder in an organic solvent [37], and subsequently applying an external field force to break the weak van der Waals forces between layers or the chemical bonds within the layers. A crucial aspect of this method is selecting an appropriate organic solvent. Due to black phosphorus’s susceptibility to oxidation in air, the selected liquid phase must be an oxygen-scavenging solvent. Common solvents for this purpose include N,N-dimethylformamide (DMF) [38], N-methyl-2-pyrrolidone (NMP) [39,40], ethanol [41], and isopropyl alcohol [42,43]. The choice of solvent also impacts the subsequent peeling and application of black phosphorus nanosheets. It is also essential to isolate the air with an inert gas during the operation to prevent the produced black phosphorus from oxidizing upon contact with air.

**Figure 5:**
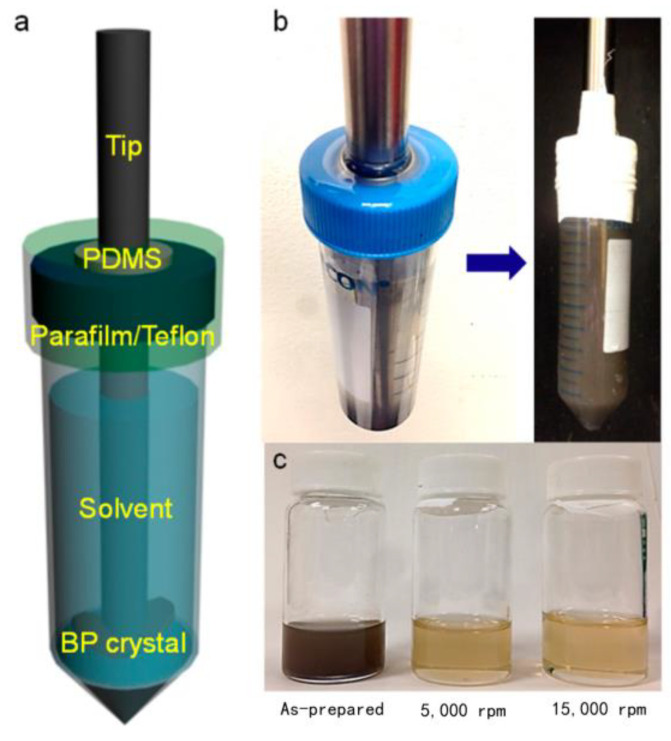
(**a**) Schematic of liquid phase stripping. (**b**) Customized tip sonication setup to minimize contact with air. (**c**) Dispersion of black phosphorus in N-methylpyrrolidone solution at various rotational speeds [44]. Reprinted with permission from Ref. [44]. Copyright 2015, copyright Joohoon Kang.

The ultrasonic exfoliation method employs ultrasonic waves to disrupt the weak van der Waals forces between the layers of black phosphorus. Given sufficient ultrasonic time and frequency, the method can even disrupt the stronger covalent bonds within the layers. The resulting black phosphorus nanosheets are smaller in size and exhibit a broader range of applications. In 2014, Kang et al. [44] successfully produced black phosphorus nanosheets using the liquid phase exfoliation method in an NMP solution (Figure 5). Guo et al. [45] subsequently added a NaOH solution to NMP to enhance its water stability, with hydroxide ions attaching to the surface of black phosphorus nanosheets. Jia [46] successfully separated black phosphorus nanosheets from an NMP solution using the liquid phase exfoliation method. Additionally, Bat-Erdene [47] found that organic solvents can isolate oxygen and prevent the oxidation of black phosphorus nanosheets. The ultrasonic peeling method partially mitigates the oxidation of black phosphorus and black phosphorene. However, during practical operation, the high boiling points of the organic solvents used result in their adsorption on the surface of the black phosphorus nanosheets, making them difficult to completely remove. This leads to the low purity of the prepared black phosphorene, thereby affecting the performance of the subsequent composite materials. Furthermore, organic solvents are harmful to the environment and human health, underscoring the importance of green organic solvents [48]. Zhao [49], Lee [50], and others discovered through extensive experimentation that ionic liquids can serve as substitutes for organic solvents. Ionic liquids are characterized by low volatility, high thermal stability, high ionic conductivity, non-toxicity, and recyclability. However, their relatively high cost may increase the overall expense of preparing black phosphorene. Chen et al. [51] substituted inexpensive deionized water for organic solvents and prepared stably dispersed black phosphorene using ultrasound. However, the efficiency of ultrasonic exfoliation alone is low. Yang et al. [52] used deoxygenated water as the solvent and applied a microwave–ultrasonic synergistic assistance method to exfoliate black phosphorus. Compared with ultrasonic exfoliation alone, this method not only increases the yield of BPNSs, but also greatly reduces the time required for exfoliation.

**Figure 6:**
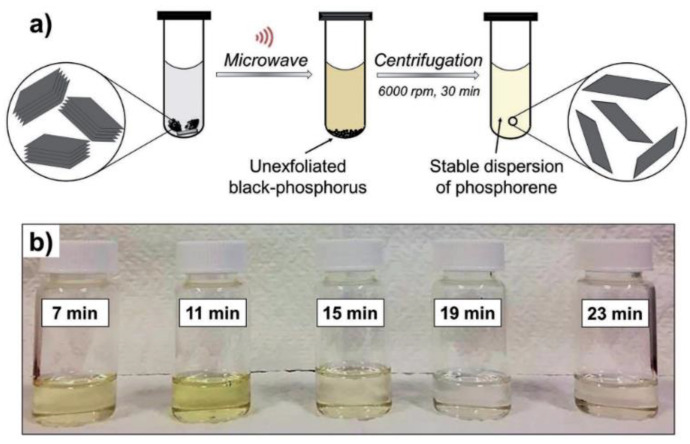
(**a**) Schematic of the MW-assisted LPE used to prepare the native BP sheet with FL-BP solution. (**b**) Photographs of the FL-BP solution in NMP prepared by stripping native BP by varying the stripping time [47]. Reprinted with permission from Ref. [47]. Copyright 2017, copyright Munkhjargal Bat-Erdene.

Microwave exfoliation is also a commonly used method to prepare two-dimensional materials. This method has been used by researchers to prepare various two-dimensional materials, including graphene and two-dimensional transition metal dichalcogenides (TMDs) [53,54]. Due to the high similarity between graphite and black phosphorene, many scholars have also attempted to use this method to prepare black phosphorus nanosheets. Wang Qin et al. [55] used NMP and dimethyl sulfoxide (DMSO) as solvents and microwave-assisted exfoliation to finally obtain black phosphorus nanosheets with a size of 40 nm × 200 nm and an average thickness of 7 nm. While this method successfully produced nanoscale black phosphorus flakes, it was time-consuming. Bat-Erdene et al. [47] successfully produced high-quality black phosphorus nanosheets in a short time by increasing the frequency of microwaves. The principle is illustrated in Figure 6. Scholars’ research indicates that the microwave exfoliation method is relatively simple and has a very short processing cycle. This method represents a novel approach to preparing black phosphorene. However, the yield of this method requires improvement. Therefore, extensive experiments are still required to investigate the effects of parameters such as time, temperature, microwave power, and other factors on the yield, size, and quality of black phosphorene. Moreover, since microwave exfoliation also involves the use of organic solvents, it is necessary to research how to identify a cost-effective and environmentally friendly organic solvent.

**Figure 7:**
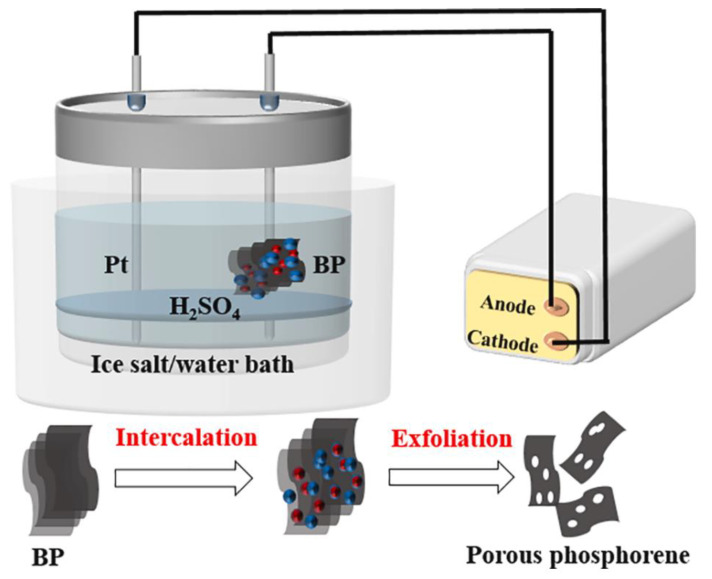
Schematic representation of black phosphorus as an anode for the synthesis of porous phosphene by the electrochemical stripping method [60]. Reprinted with permission from Ref. [60]. Copyright 2021, copyright Yuncai Jiang.

**Figure 8:**
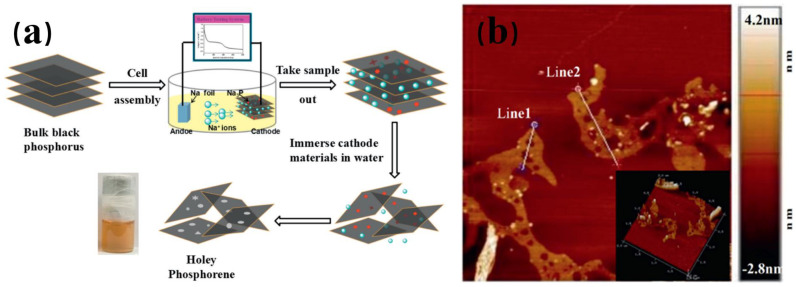
(**a**) Schematic diagram of Na3P as a cathode for the synthesis of porous black phosphazene. (**b**) AFM diagram of porous black phosphazene [62]. Reprinted with permission from Ref. [62]. Copyright 2019, copyright Honghong Liu.

The electrochemical exfoliation method was initially employed to prepare graphene and MoS_2_ with larger lateral sizes on a large scale in the early days. The fundamental principle is that the applied electric field force causes gas molecules or ions in the solution to enter the interlayer of BP, leading to an expansion in the volume of BP and consequently destroying the interlayer van der Waals force of BP. Subsequently, the required BPNSs can be obtained once the layers are separated [56,57,58]. The electrochemical stripping method can be categorized into three types based on the position of the raw material BP during the stripping process: anodic stripping, cathodic stripping, and electrolyte stripping. In 2016, Erande et al. [59] first elucidated the mechanism of the anode stripping of black phosphorus. They proposed that the attack of oxygen-containing free radicals, the insertion of ions, and the expansion of gas molecules destroyed the van der Waals force between BP layers. Jiang et al. [60] fabricated a novel type of black phosphorus nanosheet, porous black phosphorene, using the anodic stripping method. They employed BP as the anode, platinum wire as the cathode, and sulfuric acid as the electrolyte. The preparation process is shown in Figure 7. Baboukani et al. [61] utilized bipolar electrochemical exfoliation and deposition to produce BP nanosheets. Liu et al. [62] employed sodium foil as the anode and BP as the cathode to assemble a battery. The battery was discharged, placed in deoxygenated water, ultrasonicated, and centrifuged to obtain black phosphorus nanosheets. Refer to Figure 8 Atomic Force Microscope (Agilent 5500 AFM, USA).

In comparison to other the preparation methods, the electrochemical stripping method offers advantages such as environmental friendliness, efficiency, and relative controllability, making it significantly important in advancing the large-scale preparation of black phosphorene. However, this method also has two major problems that need to be urgently solved. One is its lack of controllability. Currently, the structure and size of black phosphorus nanosheets are uncontrollable, and it is not possible to produce BPNSs of the same specifications as graphene simultaneously. Secondly, the yield of black phosphorus nanosheets needs improvement. Defects in the preparation method and losses during the washing process have greatly limited the development path for large-scale preparation of black phosphorus nanosheets.

The majority of the aforementioned methods use organic solvents as the medium for stripping black phosphorus. Hence, the use of environmentally friendly organic solvents for stripping black phosphorus in the liquid phase is crucial. Additionally, while the efficiency of single ultrasonic peeling is low, the use of a synergistic method combining microwave and ultrasonic assistance not only increases the yield of BPNSs, but also significantly reduces the peeling time compared to ultrasonic peeling alone.

### 3.2. The Bottom–Up Method

#### 3.2.1. Solvothermal Method

**Figure 9:**
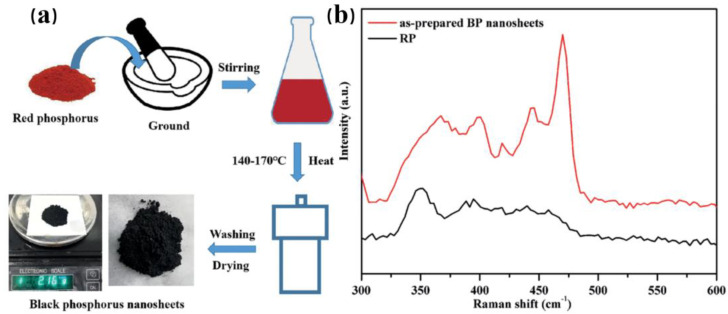
(**a**) Schematic diagram of black phosphazene synthesis from RP. (**b**) Raman spectra of the BPNSs prepared with RP [67]. Reprinted with permission from Ref. [67]. Copyright 2020, copyright Shuting Zhu.

The solvothermal method was historically used to prepare nanoparticles. It uses non-aqueous solutions or organic substances as solvents, puts the substrate into a closed space such as a reactor, and reacts by increasing the temperature and the pressure of the solution itself. The nanoparticles produced by this method have good crystal form, high purity, good dispersion, controllable particle size, low experimental temperature, and stable reaction [63]. In 2017, Zhao et al. [64] took advantage of the fact that ammonium fluoride can reduce the surface activation of the substrate. They dispersed red phosphorus (RP) powder in a solution of ammonium fluoride and distilled water, stirred it thoroughly, and placed it in a high-pressure reactor for 16 h. Finally, polycrystalline black phosphorus nanosheets were obtained after washing and drying the product. Subsequent studies have shown that bottom-up and solution-assisted routes provide a feasible strategy for the preparation of black phosphorus nanosheets under relatively mild conditions. These studies indicate that such methods are attractive because of their operational simplicity and potential cost advantages. However, the prepared black phosphorus nanosheets still face challenges such as poor crystallinity, easy oxidation, and difficulties in controlling product quality [65,66]. Similar to Tian’s scheme, Zhu [67] increased the reaction time and lowered the reaction temperature, and also prepared BPNSs by the solvothermal method (Figure 9). Subsequently, some scholars also produced porous phosphorus-based composite nanosheets by changing the solvent. These nanosheets are composed of black phosphorus, amorphous red phosphorus, and phosphorus oxide. The solvothermal method, which requires a relatively low reaction temperature, is not only simple to operate, but also cost-effective, making it widely used. However, there are also problems such as the black phosphorus nanosheets produced having a poor crystalline form and being easily oxidized.

#### 3.2.2. Chemical Vapor Deposition

The chemical vapor deposition (CVD) method refers to the reaction of one or more elemental or gas-phase compounds containing thin-film elements to form a thin film under high-temperature conditions. The nano-two-dimensional materials prepared by this method have a good crystal form and controllable size. The current technology is relatively mature and has achieved the preparation of large-area ultra-thin nano two-dimensional materials. Common materials prepared using chemical vapor deposition include graphene [68,69], transition metal sulfides [70,71], and hexagonal boron nitride (h-BN) [72,73]. In 2016, Smith et al. [74] used this method to prepare a nano-black phosphorus sheet film with an average area greater than 3 square microns and about 4 layers. Jiang et al. [75] produced a few-layer black phosphorus (non-nanoscale) using titanium foil and nanotubes as substrates under normal pressure. Izquierdo et al. [76] used red phosphorus (RP), tin, and high tin iodide as raw materials and silicon dioxide/silicon as the substrate to successfully produce micron-sized black phosphorus single crystals, but impurities were present on the surface. The biggest advantage of the chemical vapor deposition method over traditional preparation methods is that it directly skips the preparation of bulk black phosphorus and replaces it with cheap red phosphorus. The reaction time is shorter than that of traditional processes, saving a lot of costs. However, controlling the size of the produced black phosphorus film is challenging, and impurities such as white phosphorus (WP), red phosphorus (RP), and tin compounds are present on the surface of the few layers of black phosphorus. This method is still in the exploratory stage, and various technologies are not yet mature.

#### 3.2.3. High Pressure Method

**Figure 10:**
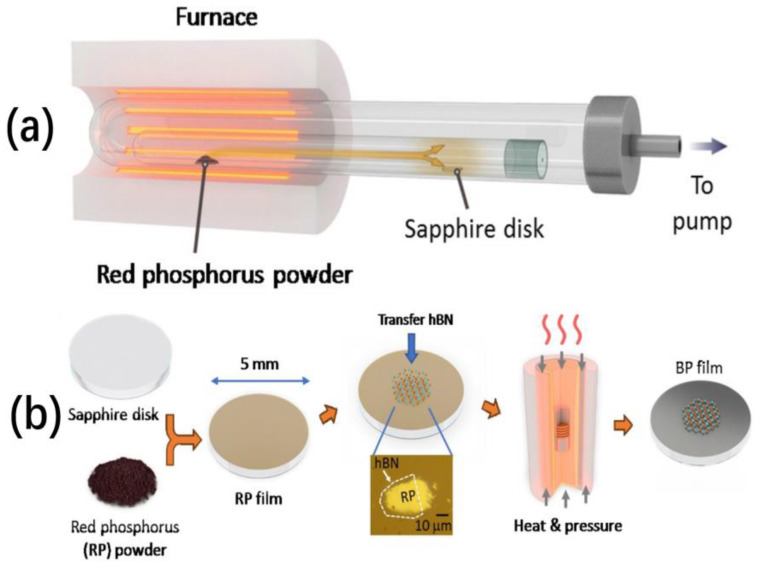
(**a**) Conversion conditions from RP to BP. (**b**) BP synthesis process flow [78]. Reprinted/adapted with permission from Ref. [78]. Copyright 2018, copyright Cheng Li.

Both black phosphorus and black phosphorene can be synthesized using high temperature and high pressure methods. The key step in synthesizing black phosphorus is applying pressure to a red phosphorus film, which undergoes a phase transformation to become black phosphorene. Li et al. [77] initially deposited a red phosphorus film on a polyethylene terephthalate (PET) substrate, followed by the application of high pressure, resulting in the formation of a black phosphorene film. Li et al. [78] deposited red phosphorus on a flexible polyester substrate under room-temperature and high-pressure conditions, thereby synthesizing a stretchable black phosphorus polycrystalline film for the first time. Subsequently, they refined the experimental procedures and synthesized a black phosphorene film on sapphire at 1.5 GPa and 700 °C (Figure 10). Black phosphorene flakes synthesized using high temperature and high pressure methods are large in size and thin, making them convenient for various applications. However, further exploration is needed for large-scale production.

### 3.3. Comparison of Various Preparation Methods

The advantages and disadvantages of various preparation methods for black phosphorus nanosheets are shown in Table 2.

## 4. Flame Retardant Mechanism and Application

### 4.1. Flame Retardant Mechanism of Black Phosphazene as Various Flame Retardants

Polymer flame retardants can be classified based on their effective elements into halogen flame retardants, nitrogen flame retardants, phosphorus flame retardants, inorganic flame retardants, etc. Halogen flame retardants release halogen compounds into the air at high temperatures. While they can inhibit combustion, they also produce toxic substances such as dioxins, bromides, and chlorides, which degrade the base material’s strength, heat resistance, and other properties [79,80,81]. Common nitrogen-based flame retardants include carbamates [82,83], phosphorus–nitrogen mixed flame retardants [84], and nitrogen heterocyclic compounds [85]. However, practical application has revealed that these flame retardants can produce nitrogen oxides and other pollutants. Their flame retardant effect is closely related to the type and shape structure of the substrate, and can sometimes affect the mechanical and conductive properties of high-performance materials (such as glass fiber, etc.). Additionally, nitrogen-based flame retardants face challenges such as difficulty in decomposition and high cost, which are also issues encountered by inorganic flame retardants.

In addition to the aforementioned flame retardants, phosphorus-based flame retardants are widely used in plastics, rubber, and electronic appliances. Phosphorus flame retardants include ether phosphate ester, ester phosphate ester, urethane phosphate ester, and nitrogen–phosphorus flame retardants [86,87]. The appropriate phosphorus flame retardant can be selected according to the specific application. Organophosphorus flame retardants, such as phosphorus oxide and phosphate esters, offer high flame retardant efficiency and low smoke volume. However, their small molecular size and poor thermal stability make them prone to migration. Inorganic phosphorus flame retardants, such as ammonium polyphosphate and red phosphorus, offer excellent thermal stability, low cost, low toxicity, and migration resistance. However, their flame retardant efficiency is significantly lower than that of organic phosphorus flame retardants.

The flame retardant mechanisms of phosphorus flame retardants include gas phase interaction, condensed phase interaction, and synergistic effects. For example, red phosphorus thermally decomposes during combustion, forming phosphoric acid, which promotes dehydration and carbonization of the polymer. This process creates a heat-resistant carbon protective layer. This protective layer isolates the substrate from contact with oxygen, slows down the generation of flammable gases, and provides heat insulation. In the initial stage of combustion, phosphorus-based flame retardants can combine with newly formed H• free radicals, OH• free radicals, and PO• free radicals to prevent free radical chain depolymerization reactions, thereby controlling combustion. Sometimes, adding a large amount of red phosphorus enhances the flame retardant effect. However, due to the large size of red phosphorus particles and their difficulty in being nano-sized, they are incompatible with the substrate and tend to be unevenly dispersed. As an allotrope of red phosphorus, black phosphorus (BP) is a chemically inert substance, which mitigates the compatibility issues. Black phosphorus’ excellent thermal stability, non-flammability, and good electrical conductivity make it widely used in electronic components, field transistors, batteries, and other applications.

**Figure 11:**
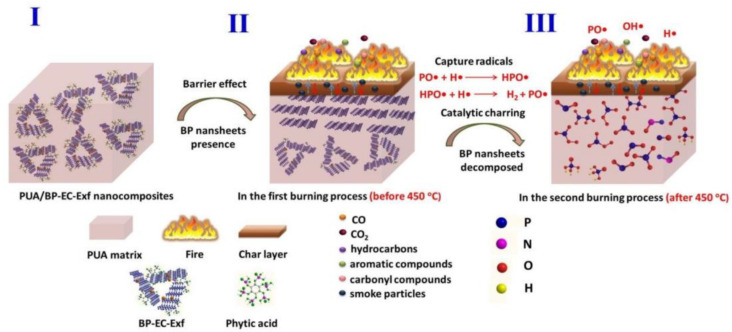
Schematic diagram of the three stages of combustion [88]. Reprinted with permission from Ref. [88]. Copyright 2019, copyright Shuilai Qiu.

The flame retardant mechanism of black phosphorus has been extensively studied by scholars. Qiu et al. [88] proposed a convincing explanation, dividing the general combustion process of the substrate into two stages (Figure 11). In the first stage, when the temperature is below 450 °C, black phosphorus nanosheets inhibit combustion through a condensed phase effect, blocking combustion by isolating flammable gases from the substrate. In the second stage, when the temperature exceeds 450 °C, black phosphorus nanosheets exhibit a dual flame retardant mechanism similar to red phosphorus, involving both condensed phase and gas phase effects [89]. BPNSs undergo thermal decomposition, generating reactive radicals such as PO•, HPO•, and PO_2_• from the undecomposed portion, which trap free radicals like H• and OH•, thereby reducing the production of combustible substances. Additionally, black phosphorus is highly susceptible to oxidation in air, leading to the formation of PxOy and other phosphoric acid derivatives. Scholars have experimentally found that epoxy resin (EP) reacts with these phosphoric acid derivatives, forming O-P-O and O-P=O complexes, which promote the formation of carbon residues. The residual carbon layer isolates the substrate from the air, inhibiting the release of heat and smoke emissions, thus achieving flame retardancy.

**Figure 12:**
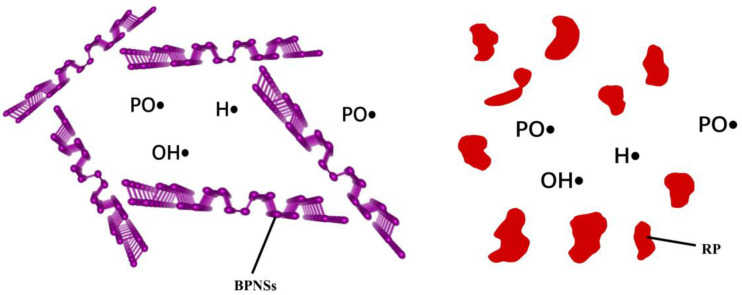
Comparison of flame retardant mechanisms of BPNSs and RP. (**On the left**) BPNSs are able to block encapsulated free radicals; (**on the right**) amorphous red phosphorus.

The flame retardant performance of a substrate is influenced by the content and size of flame retardants. BPNSs, similar to graphene, possess a lamellar structure. Micron-sized BPNSs can “encircle” free radicals such as H- and OH- to capture them, and promote the formation of a carbon layer to isolate air, thereby serving a flame retardant role (Figure 12). BPNSs at the micrometer scale can capture free radicals such as H• and OH• and promote the formation of a carbon layer to isolate air, acting as a flame retardant. In contrast, amorphous red phosphorus cannot encircle these free radicals (Figure 12). Additionally, black phosphorus exhibits a higher thermal decomposition temperature and superior mechanical properties compared to red phosphorus.

### 4.2. Black Phosphorus and Black Phosphorus Alkene for Flame Retardant Applications

#### 4.2.1. Polyurethane

**Figure 13:**
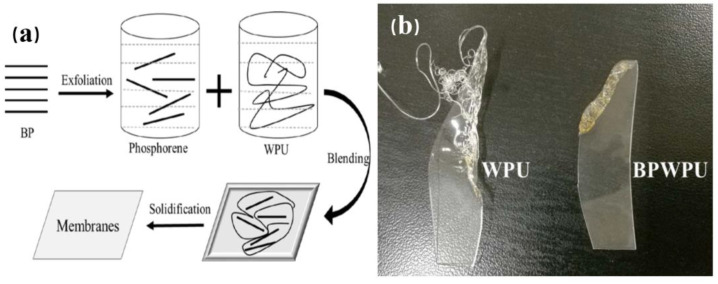
(**a**) Schematic diagram of the BP/WPU preparation process. (**b**) Burning surface images of pure WPU (**left**) and BP/WPU (**right**) strips in the surrounding environment [90]. Reprinted with permission from Ref. [90]. Copyright 2018, copyright Xinlin Ren.

Polyurethane (PU) is widely utilized in our daily lives due to its excellent mechanical properties; however, it also presents flammability challenges that need to be addressed. Ren et al. [90] used ultrasonication to obtain a black phosphorus suspension with a phosphene content of 40 mg/L, which was then mixed with waterborne polyurethane (WPU) to produce a 0.6 mm thick BP/WPU membrane. The preparation process is shown in Figure 13a. Testing showed that the LOI of BP/WPU increased from 24.2% to 26.8%, and the peak heat flow was reduced by 34.7%. Combustion experiments revealed that BP/WPU developed a significantly dense carbon layer on the surface with a residual carbon rate of 2.3%. The coke layer insulates and prevents flammable gases from reaching the substrate, enhancing the flame retardant effect. Compared to pure WPU, which produces numerous molten liquid droplets and burns out quickly, the addition of BP significantly improves the flame retardant performance of WPU. The combustion process of both materials is depicted in Figure 13b.

**Figure 14:**
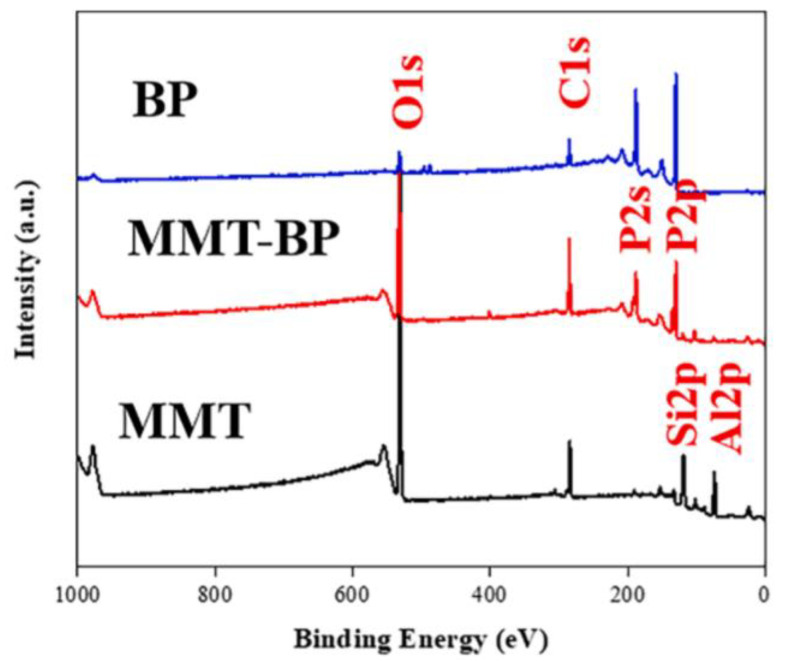
XPS measurement spectra of BP, MMT, and MMT-BP nanosheets [91]. Reprinted with permission from Ref. [91]. Copyright 2023, copyright Sihao Yin.

Yin et al. [91] prepared a montmorillonite (MMT)-modified BP through liquid phase intercalation, which was added to WPU as a flame retardant to produce the composite material MMT-BP/WPU. Experimental results showed that this composite material exhibited lower PHRR and THR compared to pure WPU, BP alone, or MMT. XPS images also indicated that the Si–O–P bond promoted the formation of a carbon layer (Figure 14). Additionally, the addition of the flame retardant did not affect the elongation at break of the composites, and the tensile strength was even increased by almost 60%.

**Figure 15:**
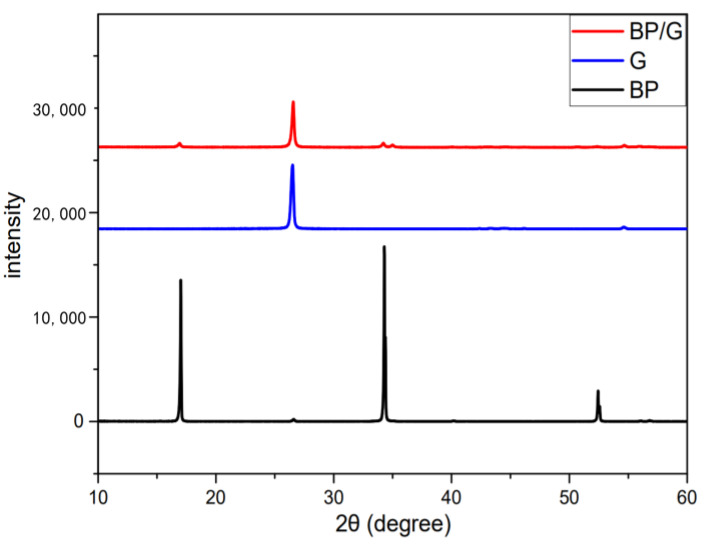
XRD images of BP/G, G, BP [92]. Reprinted with permission from Ref. [92]. Copyright 2019, copyright Xinlin Ren.

However, subsequent studies have found that BPNSs cause mechanical degradation of WPU, affecting its flame retardant effect. Ren et al. [92] prepared a new composite material, black phosphine/graphene, using a high-pressure nano-homogenizer. XRD analysis (Figure 15) showed that the characteristic peaks of black phosphine disappeared inside the composite material, indicating the breakage of the P–P bond of black phosphine to form a more stable P–C bond, thereby enhancing the environmental stability of BP.

**Figure 16:**
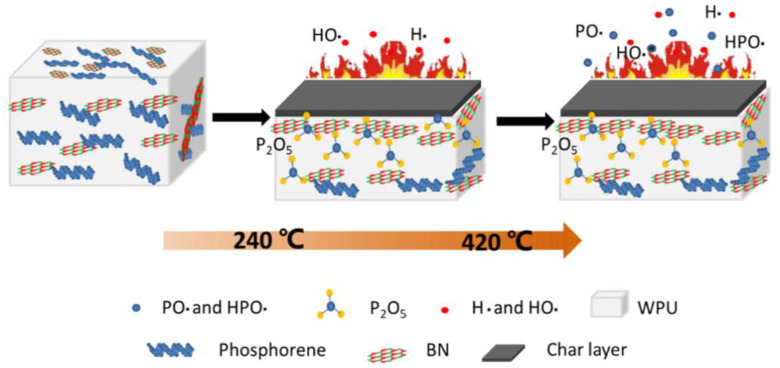
Schematic diagram of the flame retardant mechanism [95]. Reprinted with permission from Ref. [95]. Copyright 2020, copyright Sihao Yin.

Black phosphorus alone has a limited effect as a flame retardant. Some scholars have added synergistic flame retardants to enhance the flame retardant effect of black phosphorus [93,94]. Yin et al. [95] added hexagonal boron nitride (BN), another flame retardant, to the BP/WPU system to create a new BP/BN/WPU composite material. Experimental results showed that the LOI of this new composite material is as high as 33.8%, and the peak heat release rate is reduced by half, despite the flame retardant content being only 0.4 wt%. The flame retardant mechanism of this system (Figure 16) involves the combination of free radicals formed by black phosphorus, oxygen atoms, and hydrogen atoms with those generated by EP pyrolysis to inhibit the combustion chain reaction. Furthermore, the oxidized products of black phosphorus promote the formation of the carbon layer and, together with BN, isolate the contact between oxygen and the substrate.

**Figure 17:**
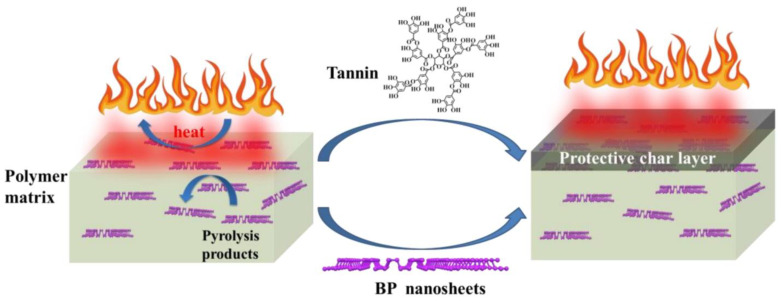
Schematic diagram of the proposed flame retardant mechanism [96]. Reprinted with permission from Ref. [96]. Copyright 2020, copyright Wei Cai.

The team at CAI [96] first prepared tannic acid (TA)-modified black phosphorus, and then used solvent blending to incorporate it into thermoplastic polyurethanes (TPU) to produce TPU/TA-BP composites. TA was chosen because it can eliminate the superoxide radicals on the surface of black phosphorus, greatly enhancing its stability, and the modified black phosphorus exhibited good dispersion within the TPU composite. The experimental results showed a 56.5% reduction in the HRR and a 43% reduction in the THR peaks of the composite (Figure 17).

Cai et al. [97] grafted polydimethylsiloxane (PDMS) onto the surface of BPNSs and incorporated it into TPU, resulting in a composite material with improved moisture resistance and flame retardant properties. Their research revealed that while PDMS enhances the environmental stability of BPNSs, BPNSs also enhance the flame retardancy of TPU. The THR and PHRR of the composite materials were reduced by 14.3% and 59.6%, respectively, and the carbon dioxide produced by combustion was also reduced by nearly half, indicating a reduction in the toxicity of the composite materials. The aforementioned studies have demonstrated that black phosphorus is well-suited for use as a flame retardant in EP.

#### 4.2.2. Epoxy Resin

Similar to polyurethane (PU), epoxy resin (EP) is characterized by excellent mechanical properties, ease of processing, and a relatively low cost, making it a popular choice in electronic appliances and transportation. However, the limited oxygen index (LOI) of this polymer is only 19.8, indicating its flammability. As a result, some researchers have incorporated black phosphorus into it to enhance its flame retardant performance.

**Figure 18:**
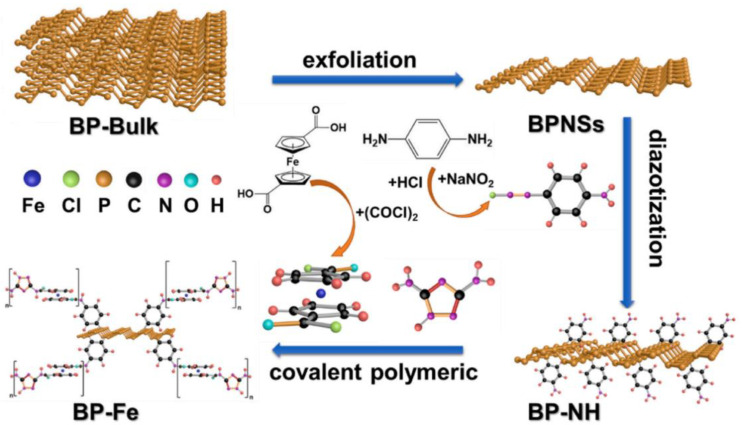
Preparation process of BP-Fe [98]. Reprinted with permission from Ref. [98]. Copyright 2022, copyright Wenhao Yang.

Yang [98] modified diazotized BP by covalently grafting ferrocene oligomers (refer to Figure 18 for the preparation process) and incorporated the modified BP into EP to enhance the strength of the composite material while suppressing smoke and reducing toxicity. Experimental results showed that with only 2 wt% modified BP added, the total smoke volume (TSP) and smoke production rate (TSP) of the EP composite decreased by about half, and the PHRR and THR decreased to 62.2% and 58.5%, respectively. Besides its significant flame retardant effect, modified BP also enhances the mechanical properties of EP composite materials and reduces the rate of organic matter volatilization, demonstrating excellent environmental stability.

**Figure 19:**
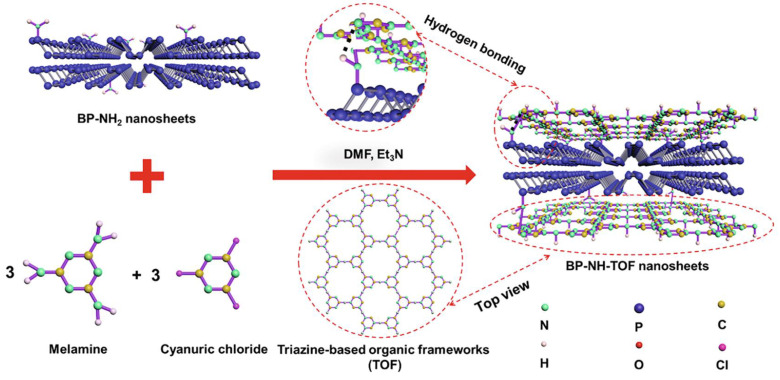
Schematic representation of the fabrication process of BP-NH-TOF nanohybrids [99]. Reprinted with permission from Ref. [99]. Copyright 2020, copyright Shuilai Qiu.

Qiu et al. [99] first discovered, through extensive data analysis, that the two-dimensional structure of covalent organic frameworks exhibits characteristics such as high crystallinity, low density, and good stability [100], making them widely used. They integrated black phosphorus with organic frameworks to explore their synergistic flame retardant effects with epoxy resins. They initially prepared aminated black phosphorus, followed by using cyanuric chloride and melamine as monomer organic framework (TOF) to grow on the surface of BPNSs via in situ polymerization, creating organic–inorganic hybrids (BP-NH-TOF). These hybrids were then incorporated into an epoxy resin matrix to produce EP composite materials (refer to Figure 19 for the preparation process). Experiments show that with the addition of only 2 wt% of BP-NH-TOF hybrid, the THR and HRR of EP composites are reduced by 44.3% and 61.2%, respectively. This method involves first aminating black phosphorus and then achieving synergistic flame retardancy. The preparation process is quite cumbersome.

**Figure 20:**
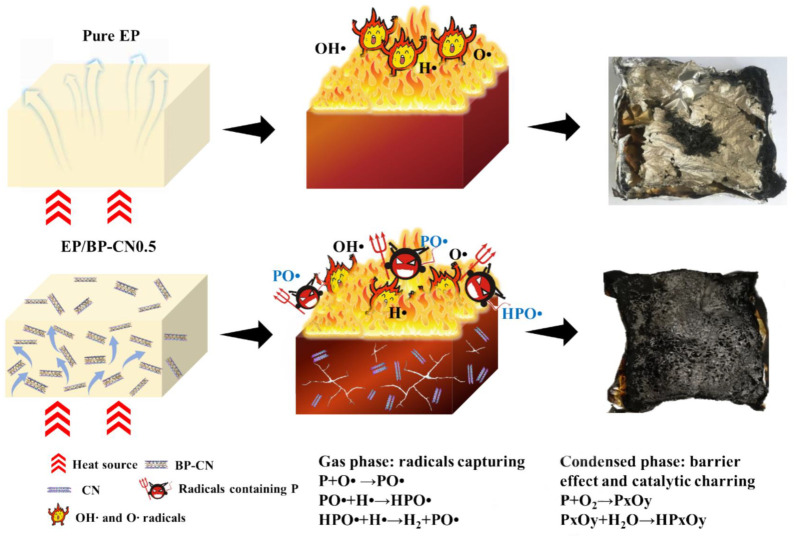
Flame retardant mechanism of EP/BP-CN nanocomposites [102]. Reprinted with permission from Ref. [102]. Copyright 2021, copyright Xiyun Ren.

The former traditional modification conditions can also obtain better results, but the actual efficiency is lower, so some scholars have improved the experimental method. Li et al. [101] utilized the electrochemical stripping method and spiral spray drying technology to prepare a novel flame retardant using BPNSs. They observed that it enhanced the flame retardancy of EP. Experimental results indicated that with a BPNS content as low as 0.94 wt%, the EP composite could achieve a V-0 rating according to UL-94. Additionally, the material exhibited a 30% improvement in mechanical properties. Ren et al. [102] employed ultrasound-assisted self-assembly of BP and graphite-like carbon nitride (g-C_3_N_4_) to synthesize a novel flame retardant, BP-CN (Figure 20), which was then incorporated into EP to produce a new composite material, EP/BP-CNx, with superior flame retardant properties. Experimental results demonstrated that the LOI of the composite material with 2 wt% BP-CNx increased to 31%, and the THR and PHRR decreased to 49.6% and 47.72%, respectively. They further adjusted the BP and CN ratio, and analysis of the carbon residue from the combustion material indicated varying fire protection properties could be achieved.

**Figure 21:**
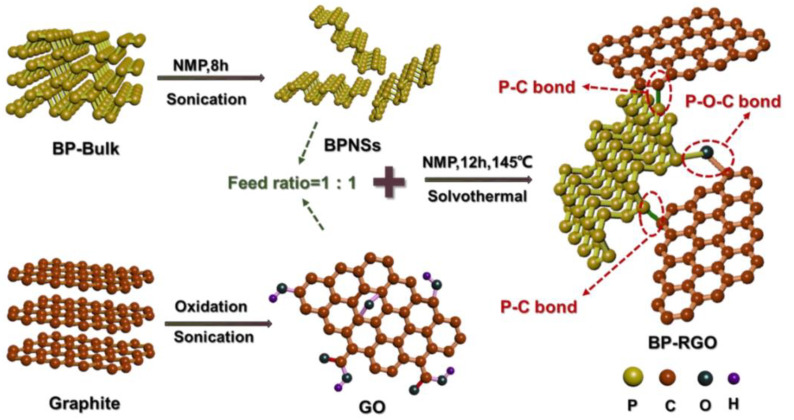
Schematic diagram for the RGO modification of BP-RGO [103]. Reprinted with permission from Ref. [103]. Copyright 2020, copyright Yifan Zhou.

Zhou et al. [103] enhanced the dispersion of BP in EP by attaching reduced graphene oxide (rGO) to its surface, creating a new flame retardant, BP-RGO nanohybrid. Experimental results showed that the PHRR and THR of EP composite materials were reduced by 55.2% and 54.4%, respectively, and the TSP was reduced by 28.5%. This indicates a significant improvement in the flame retardant performance of the EP composite material. Additionally, the EP/BP-RGO 2.0 nanocomposite exhibited good air stability after being soaked in water for a month, as discovered by the researchers (Figure 21).

**Figure 22:**
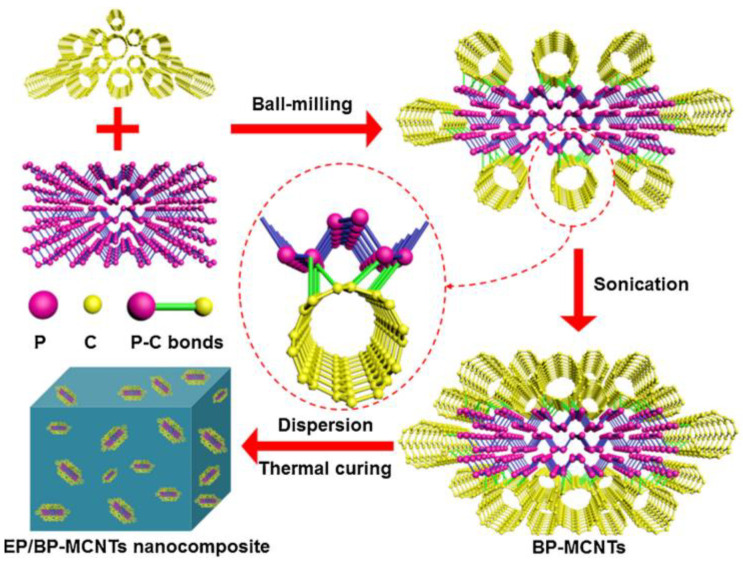
Schematic illustration for the fabrication of the BP-MCNT and its nanocomposites [104]. Reprinted with permission from Ref. [104]. Copyright 2020, copyright Bin Zou.

**Figure 23:**
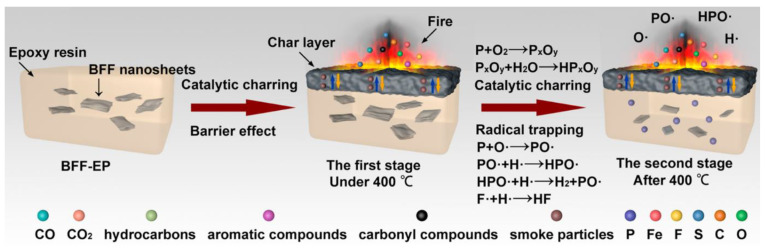
Flame retardant mechanism of BFF in EP [105]. Reprinted with permission from Ref. [105]. Copyright 2023, copyright Hanghang Chu.

Zou et al. [104] utilized a combination of liquid phase ultrasonic exfoliation and ball milling to fabricate BPCNTs from black phosphorus and multi-walled carbon nanotubes (MCNTs), which were then integrated into EP to produce new EP nanocomposites (Figure 22). The study revealed that the addition of just 2 wt% BPCNTs reduced the PHRRP and THR of the composite material by 55.81% and 41.17% respectively. TG-FTIR testing showed that the presence of numerous dense carbon layers inhibited the release of CO, indicating the significant potential of black phosphorus in enhancing the flame retardancy of polymers. Regarding the dispersion issue of black phosphorus, Chu et al. [105] proposed a new dispersion approach. They employed an iterative dispersion strategy to separate the aggregates and dispersions in the BP partial dispersion through multiple cycles to achieve an approximately uniform dispersion state. Furthermore, they utilized iron (III) trifluoromethanesulfonate functionalized black phosphorus (BFF) for flame retardant modification of the epoxy matrix (Figure 23). Experiments found that the LOI of the epoxy thermosetting plastic reached 29.2% with the addition of only 0.2 wt% BFF. However, this method also has drawbacks. It requires the use of more black phosphorus during dispersion, which increases the cost.

**Figure 24:**
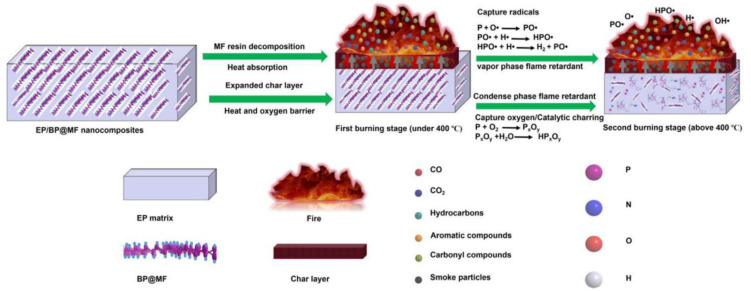
Illustration of the flame retardant mechanism of BP@MF in EP matrices [106]. Reprinted with permission from Ref. [106]. Copyright 2020, copyright Zhencai Qu.

Qu et al. [106] argued that the adsorption energy of inverted materials could influence the surface modification of BPNSs. They first calculated the adsorption energy of melamine–formaldehyde (MF) on BPNSs using density functional theory to demonstrate their compatibility. Subsequently, the MF-functionalized modified BPNSs were incorporated into the EP system to produce the new composites, resulting in an observed increase in LOI to 31.1% in experiments. Qu divided the combustion process into two phases with the 400 °C demarcation line (Figure 24). Below this temperature, the cohesive-phase flame retardant mechanism of thermal cleavage of modified BPNSs to promote the formation of a carbon layer was observed. Above this temperature, the reactive radicals and decomposed phosphoric acid derivatives generated in the process of combustion of BPNSs insulated the oxygen to achieve the flame retardant effect.

#### 4.2.3. Other Materials

**Figure 25:**
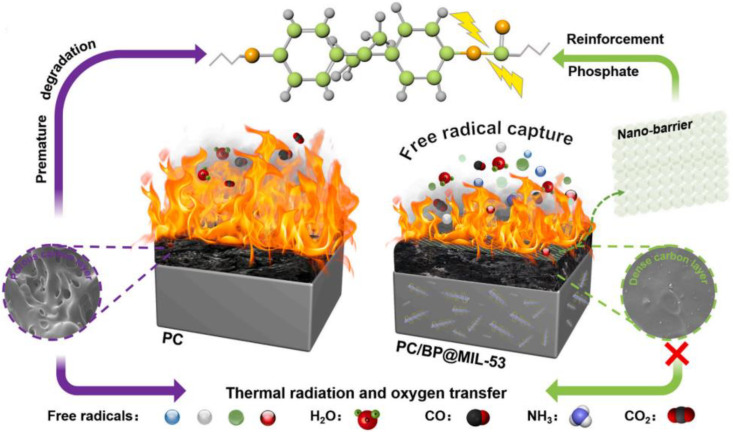
Schematic mechanism of the fire resistance and thermal stability of BP@MIL-53-reinforced PC and its nanocomposites [107]. Reprinted with permission from Ref. [107]. Copyright 2022, copyright Ziyan Qian.

Besides PU and EP, many other polymers also require improvements in their flame retardant properties. Qian et al. [107], inspired by “Cannikin’s law”, applied BPNSs with metal-organic frameworks (MOFs) to high polycarbonates (PCs) to enhance their thermal and fire safety. They first prepared BP@MIL53 hybrids from BPNSs, 2-aminobenzene dicarboxylic acid (NH_2_-BDC), and N,N-dimethylformamide (DMF) using a hydrothermal method. The flame retardant mechanism of this flame retardant mainly involves enhancing the thermal stability of PC composites by strengthening the weaker carbonate bonds. It was found that the thermal stability of PC composites with only 1 wt% of BP@MIL53 hybrids was significantly improved, and the mechanical properties of PC composites were unaffected, while the flame retardant performance was enhanced (Figure 25).

**Figure 26:**
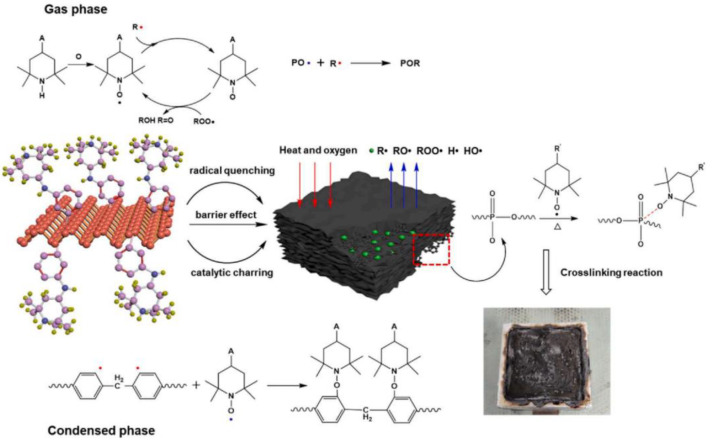
Possible flame retardant mechanism of EP/BP-HAN nanocomposites [108]. Reprinted with permission from Ref. [108]. Copyright 2020, copyright Shuilai Qiu.

Since BPNSs undergo oxidative photolysis, Qiu et al. [108] used a light stabilizer, hindered amine light stabilizer (HALS), to enhance the environmental stability of BPNSs. They first prepared negatively charged BPNSs using butyl lithium intercalation, then diazotized the black phosphorus nanosheets and grafted them with HALS. Finally, they added the modified black phosphorene to EP to obtain new composite materials (EP/BP-HAN). The characterization revealed that as the amount of modified black phosphorene increased from 0.5 wt% to 2.0 wt%, the PHRR value and THR value decreased by 32.8% and 9%, respectively (Figure 26).

**Figure 27:**
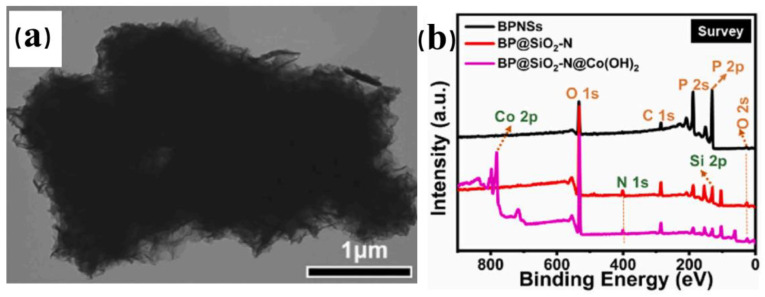
(**a**) SEM images of modified BPNSs. (**b**) XPS spectra of each material [109]. Reprinted with permission from Ref. [109]. Copyright 2023, copyright Yifan Zhou.

To reduce the toxic smoke generated by material combustion and inhibit the oxidative decomposition of black phosphorus nanosheets (BPNSs), Zhou [109] developed a novel flame retardant by encapsulating triazine-based silane and Co(OH)_2_ on the surface of BPNSs, which was subsequently added to unsaturated polyester resin (UPR). Scanning electron microscope (SEM) images indicated successful encapsulation without agglomeration, and X-ray photoelectron spectroscopy (XPS) analysis confirmed the preservation of the BPNSs’ crystal structure (Figure 27). Experimental results demonstrated a 42.3% reduction in total smoke production (TSP) for the UPR composite containing 2 wt% modified black phosphorus nanosheets.

**Figure 28:**
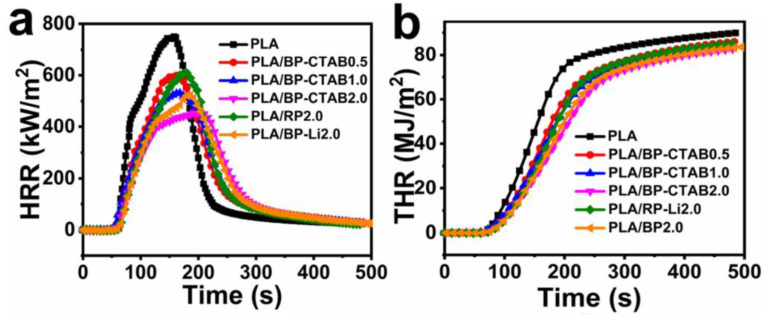
HRR (**a**) and THR (**b**) curves of PLA and its composites [110]. Reprinted with permission from Ref. [110]. Copyright 2020, copyright Yifan Zhou.

To enhance the stability of black phosphorus, Zhou et al. [110] utilized a hydrothermal reaction method to prepare modified BP functionalized with cetyltrimethylammonium bromide (CATB), which was subsequently incorporated into a polylactic acid (PLA) system to produce PLA/BP-CATB composite materials. Experimental results indicate a 24% reduction in peak heat release rate (PHRR) and a 23% reduction in total heat release (THR) with the addition of only 2 wt% BP-CATB (Figure 28).

**Figure 29:**
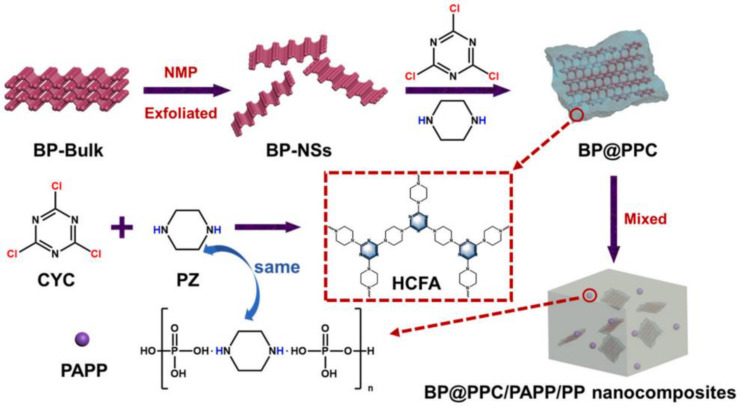
Schematic diagram for the synthesis of BP@PPC and fabrication of BP@PPC/PAPP/PP composites [113]. Reprinted with permission from Ref. [113]. Copyright 2023, copyright Wei Liu.

Moreover, some researchers have opted for environmentally friendly phytic acid to modify black phosphorus with a green approach, as phytic acid is extensively utilized in bio-based flame retardants for polymers [111,112]. Qiu’s team [88] employed electrochemical methods to fabricate cobalt phytic acid-functionalized BPNSs. Various tests revealed that these functionalized black phosphorus nanosheets not only enhanced the flame retardant properties of the composite materials but also improved their mechanical behavior. Overall, the modification of different functional groups can significantly enhance the stability and dispersion of black phosphorus (Figure 29).

Liu et al. [114] prepared BP@PPC modified with hyperbranched carbonized foaming agent (HCFA) black phosphorus through in situ polymerization and applied it to PP to enhance its flame retardant properties. In comparison to conventional piperazine pyrophosphate flame retardant (PAPP), the addition of 2 wt% BP@PPC was found to enhance the vertical combustion performance of PP to reach the V-0 level.

**Figure 30:**
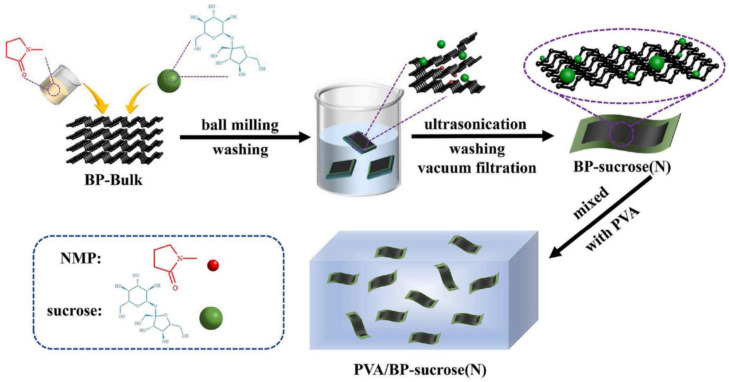
Preparation route of BP-sucrose(N) and PVA/BP-sucrose(N) [115]. Reprinted with permission from Ref. [115]. Copyright 2023, copyright Jiachen Guo.

Since black phosphorus will oxidize and decompose, Guo et al. [115] placed black phosphorus in NMP to ball-mill BPNSs. They also grafted BP with oxygen-containing groups of sucrose (N) to disperse it evenly in the PVA matrix, enhancing its air stability. Experiments found that when 5 wt% BP-N was added, the PHRR and THR of PVA composites were reduced by 52.5% and 32.8%, respectively, compared with pure PVA, and the tensile strength was increased by 131.2%. This provides a new idea for stripping BP and modifying BP (Figure 30).

Single- or few-layer black phosphorus may be a candidate material for preparing polymer nanocomposites as nanoadditives due to its mechanical properties, thermal stability, and size effects [116,117], akin to graphene [118,119]. The steps to achieve scalable exfoliation of single- or few-layer black phosphorus (BP) nanosheets are crucial for BP applications.

## 5. Summary and Vision for the Future

Black phosphorus and black phosphorus nanosheets possess broad development prospects. Their excellent performance in all aspects far surpasses that of graphene, which is currently widely used. However, the immature preparation technology and unclear formation mechanism greatly hinder the development of black phosphorus and black phosphorene.

Several major difficulties need to be addressed for black phosphorus in the future. Firstly, the preparation cost of black phosphorus is high due to its long preparation cycle and low output. Currently, most methods used in industry, academia, and research involve ultrasonic peeling, with prices typically around 2000 yuan per gram, fluctuating. Other “low-cost” methods often use dangerous chemical raw materials like white phosphorus and ethylenediamine, resulting in products that do not meet analytical purity requirements. Secondly, black phosphorus requires an oxygen-free environment for storage and experimental operations to prevent oxidation and decomposition. Some scholars have proposed a preservation method for black phosphorus similar to the one used for preserving metal aluminum, where the formation of an oxidized phosphoric acid compound on the surface of black phosphorus acts as a “separator” to isolate the air for protection. However, due to the high price of black phosphorus, this method appears to be too costly. Additionally, the modification of nano black phosphorus for flame retardant applications requires further improvement. Subsequent surface modifications can enhance its compatibility and stability with high molecular material matrices, thereby improving its flame retardant properties.

In summary, identifying a lower-cost, environmentally friendly, and high-capacity black phosphorus preparation process, along with a surface modification method to enhance its stability and compatibility, is an urgent issue that needs to be addressed.

## Figures and Tables

**Table 1:** Comparison of various preparation methods for black phosphorus.

Method	Principle	Advantages	Disadvantages
High temperature and high pressure method	High temperature and high pressure make white phosphorus and red phosphorus phase-change	Short time and easy to reproduce	Small product size, high cost, high equipment requirements
Mechanical ball milling	The high-speed impact of the ball milling medium on the red phosphorus causes it to undergo a phase change	The inert gas prevents black phosphorus from being oxidized to some extent	Poor crystal shape and time-consuming
Mercury reflux method, bismuth melting method	Mercury and bismuth are used to reduce the activation energy of the reaction	The reaction conditions are mild	Small product size, time-consuming, more polluting, and costly
Mineralization	The mineralizer reacts with red phosphorus in a series of temperature changes	Good crystallinity, high reproducibility, green environmental protection, low cost	Difficult-to-achieve scale

**Table 2:** Comparison of various preparation methods for black phosphorus nanosheets.

Method	Principle	Advantages	Disadvantages
Mechanical stripping method	Use external force to peel, such as tape, etc.	Simple operation	Time-consuming, low yield, easily oxidized
Liquid phase exfoliation	In the mixed solution with external field force, such as ultrasonic wave, microwave, electric field, etc.	The black phosphene prepared has many forms, such as BPNSs, BPQDs, porous black phosphene and so on	Higher organic solvent contamination, low yield, easily oxidized
Solvothermal method	Black phosphorus nanosheets were catalyzed by temperature, pressure and solvent in a closed system	Simple operation and low cost	Poor crystallinity, thicker black phosphorus nanosheets
Chemical vapor deposition	Nano black phosphorus was prepared by high temperature gas–solid and phase catalysis	Simple operation, low cost and not time-consuming	It is not yet mature and has impurities
High pressure method	Red phosphorus film is directly converted into black phosphorus film by applying high pressure	The black phosphorus film has a large size and thin thickness	High energy consumption and harsh reaction conditions

## Data Availability

Data are contained within the article.

## References

[1] Parasana N., Shah M., Unnarkat A. (2022). Recent advances in developing innovative sorbents for phosphorus removal-perspective and opportunities. Environ. Sci. Pollut. Res..

[2] Lambers H. (2022). Phosphorus Acquisition and Utilization in Plants. Annu. Rev. Plant Biol..

[3] Zhao M., Niu X., Guan L., Guan L., Qian H., Wang W., Sha J., Wang Y. (2016). Understanding the growth of black phosphorus crystals. CrystEngComm.

[4] Antonatos N., Bouša D., Kovalska E., Sedmidubský D., Růžička K., Vrbka P., Veselý M., Hejtmánek J., Sofer Z. (2020). Large-Scale Production of Nanocrystalline Black Phosphorus Ceramics. ACS Appl. Mater. Interfaces.

[5] Ribeiro H.B., Pimenta M.A., de Matos C.J.S. (2017). Raman spectroscopy in black phosphorus. J. Raman Spectrosc..

[6] Cheng J., Gao L., Li T., Mei S., Wang C., Wen B., Huang W., Li C., Zheng G., Wang H. (2020). Two-Dimensional Black Phosphorus Nanomaterials: Emerging Advances in Electrochemical Energy Storage Science. Nano-Micro Lett..

[7] Sui Y., Zhou J., Wang X., Wu L., Zhong S., Li Y. (2021). Recent advances in black-phosphorus-based materials for electrochemical energy storage. Mater. Today.

[8] Lee G., Kim S., Jung S., Jang S., Kim J. (2017). Suspended black phosphorus nanosheet gas sensors. Sens. Actuators B Chem..

[9] Srivastava A., Verma A., Das R., Prajapati Y.K. (2020). A theoretical approach to improve the performance of SPR biosensor using MXene and black phosphorus. Optik.

[10] Lin X., Li X., Luo B., Yu D. (2023). Black-phosphorus-based materials for application in solar cells. Chin. J. Struct. Chem..

[11] Sultana I., Rahman M.M., Ramireddy T., Chen Y., Glushenkov A.M. (2019). Correction: High capacity potassium-ion battery anodes based on black phosphorus. J. Mater. Chem. A.

[12] Han Y., Rong X., Wang M., Xue Y., Dai H., Liu Y. (2023). Progress in the preparation, application, and recycling of black phosphorus. Chemosphere.

[13] Zhou Y., Chen J., Yue L., Ren D. (2023). Preparation of High-Intensity Fluorescent Black Phosphorus Nanosheets. Phys. Status Solidi (A)—Appl. Mater. Sci..

[14] Mayorga-Martinez C.C., Sofer Z., Sedmidubský D., Luxa J., Kherzi B., Pumera M. (2018). Metallic impurities in black phosphorus nanoflakes prepared by different synthetic routes. Nanoscale.

[15] Bridgman P.W. (1935). Polymorphism, Principally of the Elements, up to 50,000 kg/cm^2^. Phys. Rev..

[16] Jacobs Robert B. (1937). Phosphorus at High Temperatures and Pressures. J. Chem. Phys..

[17] Shirotani I.J.M.C. (1982). Growth of Large Single Crystals of Black Phosphorus at High Pressures and Temperatures, and its Electrical Properties. Jpn. J. Appl. Phys..

[18] Narita S.-i., Akahama Y., Tsukiyama Y., Muro K., Mori S., Endo S., Taniguchi M., Seki M., Suga S., Mikuni A. (1983). Electrical and optical properties of black phosphorus single crystals. Physica B+C.

[19] Sun L.-Q., Li M.-J., Sun K., Yu S.-H., Wang R.-S., Xie H.-M. (2012). Electrochemical Activity of Black Phosphorus as an Anode Material for Lithium-Ion Batteries. J. Phys. Chem. C.

[20] Zhao Y.L., Yang T., Tong Y., Wang J., Luan J.H., Jiao Z.B., Chen D., Yang Y., Hu A., Liu C.T. (2017). Heterogeneous precipitation behavior and stacking-fault-mediated deformation in a CoCrNi-based medium-entropy alloy. Acta Mater..

[21] Günther P.L., Gesslle P., Rebentisch W. (1943). Untersuchungen zum Diamantproblem. Z. Anorg. Allg. Chem..

[22] Park C.-M., Sohn H.J. (2007). Black Phosphorus and its Composite for Lithium Rechargeable Batteries. Adv. Mater..

[23] Nagao M., Hayashi A., Tatsumisago M. (2010). All-solid-state lithium secondary batteries with high capacity using black phosphorus negative electrode. J. Power Sources.

[24] Krebs H., Weitz H.P., Worms K.H. (1955). Über die Struktur und Eigenschaften der Halbmetalle. VIII. Die katalytische Darstellung des schwarzen Phosphors. Z. Anorg. Allg. Chem..

[25] Baba M., Izumida F., Takeda Y., Morita A. (1989). Preparation of Black Phosphorus Single Crystals by a Completely Closed Bismuth-Flux Method and Their Crystal Morphology. Jpn. J. Appl. Phys..

[26] Lange S., Schmidt P., Nilges T. (2007). Au3SnP7@black phosphorus: An easy access to black phosphorus. Inorg. Chem..

[27] Nilges T., Kersting M., Pfeifer T. (2008). A fast low-pressure transport route to large black phosphorus single crystals. J. Solid State Chem..

[28] Köpf M., Eckstein N., Pfister D., Grotz C., Krüger I., Greiwe M., Hansen T., Kohlmann H., Nilges T. (2014). Access and in situ growth of phosphorene-precursor black phosphorus. J. Cryst. Growth.

[29] Li S., Liu X., Fan X., Ni Y., Miracle J., Theodoropoulou N., Sun J., Chen S., Lv B., Yu Q.J.C.G. (2017). Addition to New Strategy for Black Phosphorus Crystal Growth through Ternary Clathrate. Cryst. Growth Des..

[30] Novoselov K.S., Geim A.K., Morozov S.V., Jiang D., Zhang Y., Dubonos S.V., Grigorieva I.V., Firsov A.A. (2004). Electric field effect in atomically thin carbon films. Science.

[31] Li L., Yu Y., Ye G.J., Ge Q., Ou X., Wu H., Feng D., Chen X.H., Zhang Y. (2014). Black phosphorus field-effect transistors. Nat. Nanotechnol..

[32] Chen Y., Jiang G., Chen S., Guo Z., Yu X., Zhao C., Zhang H., Bao Q., Wen S., Tang D. (2015). Mechanically exfoliated black phosphorus as a new saturable absorber for both Q-switching and Mode-locking laser operation. Optics Express.

[33] Castellanos-Gomez A., Vicarelli L., Prada E., Island J.O., Narasimha-Acharya K.L., Blanter S.I., Groenendijk D.J., Buscema M., Steele G.A., Álvarez J.A.V. (2014). Isolation and characterization of few-layer black phosphorus. 2D Mater..

[34] Lu W., Nan H., Hong J., Chen Y., Zhu C., Liang Z., Ma X., Ni Z., Jin C., Zhang Z. (2014). Plasma-assisted fabrication of monolayer phosphorene and its Raman characterization. Nano Res..

[35] Buscema M., Groenendijk D.J., Blanter S.I., Steele G.A., van der Zant H.S., Castellanos-Gomez A. (2014). Fast and Broadband Photoresponse of Few-Layer Black Phosphorus Field-Effect Transistors. Nano Lett..

[36] Hu B., Zhang T., Wang K., Wang L., Zhang Y., Gao S., Ye X., Zhou Q., Jiang S., Li X. (2023). Narrow Directed Black Phosphorus Nanoribbons Produced by A Reformative Mechanical Exfoliation Approach. Small.

[37] Cao Y., Tian X., Gu J., Liu B., Zhang B., Song S., Fan F., Chen Y.J.A.C. (2018). Covalent Functionalization of Black Phosphorus with Conjugated Polymer for Information Storage. Angew. Chem..

[38] Shen J., Liu L., Huang W., Wu K. (2021). Polyvinylpyrrolidone-assisted solvent exfoliation of black phosphorus nanosheets and electrochemical sensing of p-nitrophenol. Anal. Chim. Acta.

[39] Ma J., Osei Lartey P., Guo K., Liu J., Ren M., Zhao Y., Yang Y. (2020). Exfoliating two-dimensional materials into few layers via optimized environmentally-friendly ternary solvents. Nanotechnology.

[40] Tiouitchi G., Ali M.A., Benyoussef A., Hamedoun M., Lachgar A., Kara A., Ennaoui A., Mahmoud A., Boschini F., Oughaddou H. (2020). Efficient production of few-layer black phosphorus by liquid-phase exfoliation. R. Soc. Open Sci..

[41] Du K., Yang W., Deng S., Li X., Yang P. (2020). High-Quality Black Phosphorus Quantum Dots Fabricated via Microwave-Tailored Technology. Nanomaterials.

[42] Bartus C.P., Hegedűs T., Kozma G., Szenti I., Vajtai R., Kónya Z., Kukovecz Á. (2022). Exfoliation of black phosphorus in isopropanol-water cosolvents. J. Mol. Struct..

[43] Jia J., Jeon S., Jeon J., Park J.-H., Lee S. (2019). Versatile Doping Control of Black Phosphorus and Functional Junction Structures. J. Phys. Chem. C.

[44] Kang J., Wood J.D., Wells S.A., Lee J.H., Liu X., Chen K.S., Hersam M.C. (2015). Solvent Exfoliation of Electronic-Grade, Two-Dimensional Black Phosphorus. ACS Nano.

[45] Guo Z., Zhang H., Lu S., Wang Z., Tang S., Shao J., Sun Z., Xie H., Wang H., Yu X.-F. (2015). From Black Phosphorus to Phosphorene: Basic Solvent Exfoliation, Evolution of Raman Scattering, and Applications to Ultrafast Photonics. Adv. Funct. Mater..

[46] Jia C., Zhao L., Cui M., Yang F., Cheng G., Yang G., Zeng Z. (2019). Surface coordination modification and electrical properties of few-layer black phosphorus exfoliated by the liquid-phase method. J. Alloys Compd..

[47] Bat-Erdene M., Batmunkh M., Shearer C.J., Tawfik S.A., Ford M.J., Yu L.-Y., Sibley A., Slattery A.D., Quinton J.S., Gibson C.T. (2017). Efficient and Fast Synthesis of Few-Layer Black Phosphorus via Microwave-Assisted Liquid-Phase Exfoliation. Small Methods.

[48] Tobiszewski M., Namieśnik J. (2017). Greener organic solvents in analytical chemistry. Curr. Opin. Green Sustain. Chem..

[49] Zhao W., Xue Z., Wang J., Jiang J., Zhao X., Mu T. (2015). Large-Scale, Highly Efficient, and Green Liquid-Exfoliation of Black Phosphorus in Ionic Liquids. ACS Appl. Mater. Interfaces.

[50] Lee M., Roy A.K., Jo S., Choi Y., Chae A., Kim B., Park S.Y., In I. (2017). Exfoliation of black phosphorus in ionic liquids. Nanotechnology.

[51] Chen L., Zhou G., Liu Z., Ma X., Chen J., Zhang Z., Ma X., Li F., Cheng H.M., Ren W. (2016). Scalable Clean Exfoliation of High-Quality Few-Layer Black Phosphorus for a Flexible Lithium Ion Battery. Adv. Mater..

[52] Yang Y., Chen X., Lian P., Chen R., Liu Y., Mei Y. (2018). Production of Phosphorene from Black Phosphorus via Sonication and Microwave Co-assisted Aqueous Phase Exfoliation. Chem. Lett..

[53] Kim H.-R., Lee S.-H., Lee K.-H. (2018). Scalable production of large single-layered graphenes by microwave exfoliation ‘in deionized water’. Carbon.

[54] Feng L., Zhao D., Yu J., Zhao Q., Yuan X., Liu Y., Guo S. (2023). Two-dimensional transition metal dichalcogenides based composites for microwave absorption applications: A review. J. Phys. Energy.

[55] Wang Q., Yu X., Fu M., Deng S., Feng X., Yang W., Yang P. (2022). Synthesis and antioxidation of low-dimensional black phosphorus. Acta Opt. Sin..

[56] Carrasco D.F., Paredes J.I., Villar-Rodil S., Suárez-García F., Martínez-Alonso A., Tascón J.M.D.J.C. (2022). An electrochemical route to holey graphene nanosheets for charge storage applications. Carbon.

[57] Zhou A., Bai J., Hong W., Bai H. (2022). Electrochemically reduced graphene oxide: Preparation, composites, and applications. Carbon.

[58] Guo T., Wang L., Sun S., Wang Y., Chen X., Zhang K., Zhang D., Xue Z., Zhou X. (2019). Layered MoS_2_@graphene functionalized with nitrogen-doped graphene quantum dots as an enhanced electrochemical hydrogen evolution catalyst. Chin. Chem. Lett..

[59] Erande M.B., Pawar M.S., Late D.J. (2016). Humidity Sensing and Photodetection Behavior of Electrochemically Exfoliated Atomically Thin-Layered Black Phosphorus Nanosheets. ACS Appl. Mater. Interfaces.

[60] Jiang Y., Hou R., Lian P., Fu J., Lu Q., Mei Y. (2021). A facile and mild route for the preparation of holey phosphorene by low-temperature electrochemical exfoliation. Electrochem. Commun..

[61] Rabiei Baboukani A., Khakpour I., Drozd V., Allagui A., Wang C. (2019). Single-step exfoliation of black phosphorus and deposition of phosphorene via bipolar electrochemistry for capacitive energy storage application. J. Mater. Chem. A.

[62] Liu H., Lian P., Zhang Q., Yang Y., Mei Y. (2019). The preparation of holey phosphorene by electrochemical assistance. Electrochem. Commun..

[63] Mousavi S.H., Müller T.S., Karos R., de Oliveira P.W. (2016). Faster synthesis of CIGS nanoparticles using a modified solvothermal method. J. Alloys Compd..

[64] Zhao G., Wang T., Shao Y., Wu Y., Huang B., Hao X. (2017). A Novel Mild Phase-Transition to Prepare Black Phosphorus Nanosheets with Excellent Energy Applications. Small.

[65] Gusmão R., Sofer Z., Pumera M. (2017). Black phosphorus rediscovered: From bulk material to monolayers. Angew. Chem. Int. Ed..

[66] Akhtar M., Anderson G., Zhao R., Alruqi A., Mroczkowska J.E., Sumanasekera G., Jasinski J.B. (2017). Recent advances in synthesis, properties, and applications of phosphorene. npj 2D Mater. Appl..

[67] Zhu S., Liang Q., Xu Y., Fu H., Xiao X.J.E.J.o.I.C. (2020). Facile Solvothermal Synthesis of Black Phosphorus Nanosheets from Red Phosphorus for Efficient Photocatalytic Hydrogen Evolution. Eur. J. Inorg. Chem..

[68] Li X., Colombo L., Ruoff R.S. (2016). Synthesis of Graphene Films on Copper Foils by Chemical Vapor Deposition. Adv. Mater..

[69] Cai X., Wen S., Lv B., Dou W.-D. (2023). Vertical-Graphene-Assisted Chemical Vapor Deposition for Fast Growth of Macroscaled Graphene Grains. J. Phys. Chem. C.

[70] Li S., Ouyang D., Zhang N., Zhang Y., Murthy A., Li Y., Liu S., Zhai T. (2023). Substrate Engineering for Chemical Vapor Deposition Growth of Large-Scale 2D Transition Metal Dichalcogenides. Adv. Mater..

[71] Wang J., Huang J., Li Y., Ding K., Jiang D., Dou X., Sun B. (2022). Radiative and Non-Radiative Exciton Recombination Processes in a Chemical Vapor Deposition-Grown MoSe2 Film. J. Phys. Chem. C.

[72] Feng Y., Zhang Y., Liu J., Zhang Y., Xie Y. (2022). Large-Scale Synthesis h-BN Films on Copper-Nickel Alloy by Atmospheric Pressure Chemical Vapor Deposition. Crystals..

[73] Saji V.S. (2023). 2D hexagonal boron nitride (h-BN) nanosheets in protective coatings: A literature review. Heliyon.

[74] Smith J.B., Hagaman D., Ji H.F. (2016). Growth of 2D black phosphorus film from chemical vapor deposition. Nanotechnology.

[75] Jiang Q., Xu L., Chen N., Zhang H., Dai L., Wang S. (2016). Facile Synthesis of Black Phosphorus: An Efficient Electrocatalyst for the Oxygen Evolving Reaction. Angew. Chem. Int. Ed..

[76] Izquierdo N., Myers J.C., Seaton N.C., Pandey S.K., Campbell S.A. (2019). Thin-Film Deposition of Surface Passivated Black Phosphorus. ACS Nano.

[77] Li X., Deng B., Wang X., Chen S., Vaisman M., Karato S.-i., Pan G., Larry Lee M., Cha J., Wang H. (2015). Synthesis of thin-film black phosphorus on a flexible substrate. 2D Mater..

[78] Li C., Wu Y.Q., Deng B., Xie Y., Guo Q., Yuan S., Chen X., Bhuiyan M.A., Wu Z., Watanabe K. (2018). Synthesis of Crystalline Black Phosphorus Thin Film on Sapphire. Adv. Mater..

[79] Bevington C., Williams A.J., Guider C., Baker N.C., Meyer B., Babich M.A., Robinson S., Jones A., Phillips K.A. (2022). Development of a Flame Retardant and an Organohalogen Flame Retardant Chemical Inventory. Sci. Data.

[80] Appavoo D., Amarnath N., Lochab B. (2020). Cardanol and Eugenol Sourced Sustainable Non-halogen Flame Retardants for Enhanced Stability of Renewable Polybenzoxazines. Front. Chem..

[81] Cai T., Wang J., Zhang C., Cao M., Jiang S., Wang X., Wang B., Hu W., Hu Y. (2020). Halogen and halogen-free flame retarded biologically-based polyamide with markedly suppressed smoke and toxic gases releases. Compos. Part B Eng..

[82] Passauer L. (2019). Thermal characterization of ammonium starch phosphate carbamates for potential applications as bio-based flame-retardants. Carbohydr. Polym..

[83] Luda M.P., Costa L., Bracco P., Levchik S.V. (2004). Relevant factors in scorch generation in fire retarded flexible polyurethane foams: II Reactivity of isocyanate, urea and urethane groups. Polym. Degrad. Stab..

[84] Deng C., Ji Y., Zhu M., Liang Y., Jian H., Yan Z., Wen M., Park H. (2023). Preparation of Organic-Inorganic Phosphorus-Nitrogen-Based Flame Retardants and Their Application to Plywood. Polymers.

[85] Lu S., Chen S., Luo L., Yang Y., Wang J., Chen Y., Yang Y., Yuan Z., Chen X. (2023). Molecules Featuring the Azaheterocycle Moiety toward the Application of Flame-Retardant Polymers. ACS Chem. Health Saf..

[86] Krumm E.A., Patel V.J., Tillery T.S., Yasrebi A., Shen J., Guo G.L., Marco S.M., Buckley B.T., Roepke T.A. (2017). Organophosphate Flame-Retardants Alter Adult Mouse Homeostasis and Gene Expression in a Sex-Dependent Manner Potentially Through Interactions With ERα. Toxicol. Sci..

[87] Cheng X.-W., Guan J.-P., Tang R.-C., Liu K.-Q. (2016). Phytic acid as a bio-based phosphorus flame retardant for poly(lactic acid) nonwoven fabric. J. Clean. Prod..

[88] Qiu S., Zou B., Sheng H., Guo W., Wang J., Zhao Y., Wang W., Yuen R.K.K., Kan Y., Hu Y. (2019). Electrochemically Exfoliated Functionalized Black Phosphorene and Its Polyurethane Acrylate Nanocomposites: Synthesis and Applications. ACS Appl. Mater. Interfaces.

[89] Qiu S., Zhou Y., Zhou X., Zhang T., Wang C., Yuen R.K.K., Hu W., Hu Y. (2019). Air-Stable Polyphosphazene-Functionalized Few-Layer Black Phosphorene for Flame Retardancy of Epoxy Resins. Small.

[90] Ren X., Mei Y., Lian P., Xie D., Yang Y., Wang Y., Wang Z. (2018). A Novel Application of Phosphorene as a Flame Retardant. Polymers.

[91] Yin S., Ren X., Zheng R.X., Li Y., Zhao J., Xie D., Mei Y.J.C.E.J. (2023). Improving fire safety and mechanical properties of waterborne polyurethane by montmorillonite-passivated black phosphorus. Chem. Eng. J..

[92] Ren X., Mei Y., Lian P., Xie D., Deng W., Wen Y., Luo Y.J.P. (2019). Fabrication and Application of Black Phosphorene/Graphene Composite Material as a Flame Retardant. Polymers.

[93] Zhang T., Xie H., Xie S., Hu A., Liu J., Kang J., Hou J., Hao Q., Liu H., Ji H. (2023). A Superior Two-Dimensional Phosphorus Flame Retardant: Few-Layer Black Phosphorus. Molecules.

[94] Li J., Wu J., Wei X., Yu Q., Han Y., Yu J., Wang Z. (2022). High-Performance TPE-S Modified by a Flame-Retardant System Based on Black Phosphorus Nanosheets. ACS Omega.

[95] Yin S., Ren X., Lian P., Zhu Y., Mei Y.J.P. (2020). Synergistic Effects of Black Phosphorus/Boron Nitride Nanosheets on Enhancing the Flame-Retardant Properties of Waterborne Polyurethane and Its Flame-Retardant Mechanism. Polymers.

[96] Cai W., Cai T., He L., Chu F., Mu X., Han L., Hu Y., Wang B., Hu W. (2020). Natural antioxidant functionalization for fabricating ambient-stable black phosphorus nanosheets toward enhancing flame retardancy and toxic gases suppression of polyurethane. J. Hazard. Mater..

[97] Cai W., Mu X., Li Z., Hu W., Hu Y. (2022). Poly(dimethyl siloxane)-grafted black phosphorus nanosheets as filler to enhance moisture-resistance and flame-retardancy of thermoplastic polyurethane. Mater. Chem. Phys..

[98] Yang W., Qiu S., Zhou Y., Wang J., Zou B., Song L. (2022). Covalent grafting diazotized black phosphorus with ferrocene oligomer towards smoke suppression and toxicity reduction. Chemosphere.

[99] Qiu S., Zou B., Zhang T., Ren X., Yu B., Zhou Y., Kan Y., Hu Y. (2020). Integrated effect of NH2-functionalized/triazine based covalent organic framework black phosphorus on reducing fire hazards of epoxy nanocomposites. Chem. Eng. J..

[100] Chen Z., Li X., Yang C., Cheng K., Tan T., Lv Y., Liu Y. (2021). Hybrid Porous Crystalline Materials from Metal Organic Frameworks and Covalent Organic Frameworks. Adv. Sci..

[101] Li C., Cui X., Gao X., Liu S., Sun Q., Lian H., Zu L., Liu Y., Wang X., Cui X.J.C.I. (2020). Electrochemically prepared black phosphorene micro-powder as flame retardant for epoxy resin. Compos. Interfaces.

[102] Ren X., Zou B., Zhou Y., Zhao Z., Qiu S., Song L. (2021). Construction of few-layered black phosphorus/graphite-like carbon nitride binary hybrid nanostructure for reducing the fire hazards of epoxy resin. J. Coll. Interface Sci..

[103] Zhou Y., Chu F., Qiu S., Guo W., Zhang S., Xu Z., Hu W., Hu Y. (2020). Construction of graphite oxide modified black phosphorus through covalent linkage: An efficient strategy for smoke toxicity and fire hazard suppression of epoxy resin. J. Hazard. Mater..

[104] Zou B., Qiu S., Ren X., Zhou Y., Zhou F., Xu Z., Zhao Z., Song L., Hu Y., Gong X. (2020). Combination of black phosphorus nanosheets and MCNTs via phosphoruscarbon bonds for reducing the flammability of air stable epoxy resin nanocomposites. J. Hazard. Mater..

[105] Chu H., Wu M., Liu X., Liu X., Pan J., Liu S., Zhao J., Xie W.J.A.A.N.M. (2023). Uniformly-Dispersed Black Phosphorene as Flame-Retardant Epoxy Composites via Iterative Dispersion Strategy. ACS Appl. Nano Mater..

[106] Qu Z., Wu K., Jiao E., Chen W., Hu Z., Xu C., Shi J., Wang S., Tan Z. (2020). Surface functionalization of few-layer black phosphorene and its flame retardancy in epoxy resin. Chem. Eng. J..

[107] Qian Z., Zou B., Xiao Y., Qiu S., Xu Z., Yang Y., Jiang G., Zhang Z., Song L., Hu Y. (2022). Targeted modification of black phosphorus by MIL-53(Al) inspired by “Cannikin’s Law” to achieve high thermal stability of flame retardant polycarbonate at ultra-low additions. Compos. Part B Eng..

[108] Qiu S., Hou Y., Zhou X., Zhou Y., Wang J., Zou B., Yang W., Song L., Hu Y.J.C.E.J. (2021). Light stabilizer and diazo passivation of black phosphorus nanosheets: Covalent functionalization endows air stability and flame retadancy enhancements. Chem. Eng. J..

[109] Zhou Y., Qiu S., Chu F., Liu W., Yang W., Hu W., Hu Y. (2023). Passivation of black phosphorus by triazine-based silica coating: Hierarchical BP@SiO_2_-N@Co(OH)_2_ structure for enhanced fire safety and toughness of unsaturated polyester resins. Compos. Part A Appl. Sci. Manufact..

[110] Zhou Y., Huang J., Wang J., Chu F., Xu Z., Hu W., Hu Y. (2020). Rationally designed functionalized black phosphorus nanosheets as new fire hazard suppression material for polylactic acid. Polym. Degrad. Stab..

[111] Shang S., Yuan B., Sun Y., Chen G., Huang C., Yu B., He S., Dai H., Chen X. (2019). Facile preparation of layered melamine-phytate flame retardant via supramolecular self-assembly technology. J. Coll. Interface Sci..

[112] Cheng X.-W., Guan J.-P., Yang X.-H., Tang R.-C. (2017). Improvement of flame retardancy of silk fabric by bio-based phytic acid, nano-TiO2, and polycarboxylic acid. Prog. Org. Coat..

[113] Liu W., Ding L., Wang L., Zhang C., Yang W., Liu D., Gui Z., Hu W.J.C. (2023). A rational design of functionalized black phosphorus cooperates with piperazine pyrophosphate to significantly suppress the fire hazards of polypropylene. Chemosphere.

[114] Liu T., Lu Y., Zhan R., Qian W., Luo G. (2023). Nanomaterials and nanomaterials-based drug delivery to promote cutaneous wound healing. Adv. Drug Deliv. Rev..

[115] Guo J., Yang L., Zhang L., Li C. (2022). Simultaneous exfoliation and functionalization of black phosphorus by sucrose-assisted ball milling with NMP intercalating and preparation of flame retardant polyvinyl alcohol film. Polymer.

[116] Zou B., Qiu S., Zhou Y., Qian Z., Xu Z., Wang J., Xiao Y., Liao C., Yang W., Han L. (2022). Photothermal-healing, and record thermal stability and fire safety black phosphorus–boron hybrid nanocomposites: Mechanism of phosphorus fixation effects and charring inspired by cell walls. J. Mater. Chem. A.

[117] McDowell M.T., Xiong H., Nazemi M., Peng J., Lutkenhaus J.L., Wang R., Djire A., Sankaran A., Leem J., Gogotsi Y. (2023). Nanomaterials in the future of energy research. Cell Rep. Phys. Sci..

[118] Liu Q., Zhao Y., Gao S., Yang X., Fan R., Zhi M., Fu M. (2021). Recent advances in the flame retardancy role of graphene and its derivatives in epoxy resin materials. Compos. Part A Appl. Sci. Manufact..

[119] Ge Q., Chu J., Cao W., Yi F., Ran Z., Jin Z., Mao B., Li Z., Novoselov K.S.J.A.F.M. (2022). Graphene-Based Textiles for Thermal Management and Flame Retardancy. Adv. Funct. Mater..

