# Correction: Cao et al. Research Progress on the Preparation Methods for and Flame Retardant Mechanism of Black Phosphorus and Black Phosphorus Nanosheets. *Nanomaterials* 2024, *14*, 892

**DOI:** 10.3390/nano16070421

**Published:** 2026-03-31

**Authors:** Wuyan Cao, Dengwang Lai, Jun Yang, Li Liu, Hao Wu, Jin Wang, Yuejun Liu

**Affiliations:** 1 Key Laboratory of Advanced Packaging Materials and Technology of Hunan Province, Hunan University of Technology, Zhuzhou 412007, China; cwy459980243@163.com (W.C.); m23080500012@stu.hut.edu.cn (L.L.); m21080500014@stu.hut.edu.cn (H.W.); liuyuejun@hut.edu.cn (Y.L.); 2 Zhuzhou Times New Material Technology Co., Ltd., Zhuzhou 412007, China; wangjin@csrzic.com

In the original publication [1], reference [65] was retracted prior to this publication. Concerns were raised on this matter. For scientific consistency and to avoid potential bias, the authors would like to replace reference 65 and reference 66. 

65. Gusmão, R.; Sofer, Z.; Pumera, M. Black phosphorus rediscovered: From bulk material to monolayers. *Angew. Chem. Int. Ed.* **2017**, *56*, 8052–8072.

66. Akhtar, M.; Anderson, G.; Zhao, R.; Alruqi, A.; Mroczkowska, J.E.; Sumanasekera, G.; Jasinski, J.B. Recent advances in synthesis, properties, and applications of phosphorene. *npj 2D Mater. Appl.* **2017**, *1*, 5.

Due to the replacement of references, the corresponding citations and sentences in Section 3.2.1 have been adjusted accordingly to align with numerical order. Also, due to updates to the main text, Figure 9 and its citations have been removed, and the numbering and citations of the subsequent figures have been adjusted accordingly.

“Subsequent studies have shown that bottom-up and solution-assisted routes provide a feasible strategy for the preparation of black phosphorus nanosheets under relatively mild conditions. These studies indicate that such methods are attractive because of their operational simplicity and potential cost advantages. However, the prepared black phosphorus nanosheets still face challenges such as poor crystallinity, easy oxidation, and difficulties in controlling product quality [65,66].”

The authors state that the scientific conclusions are unaffected. This correction was approved by the Academic Editor. The original publication has also been updated.
